# Guidelines for laparoscopic treatment of ventral and incisional abdominal wall hernias (International Endohernia Society [IEHS])—Part 2

**DOI:** 10.1007/s00464-013-3171-5

**Published:** 2013-11-14

**Authors:** R. Bittner, J. Bingener-Casey, U. Dietz, M. Fabian, G. S. Ferzli, R. H. Fortelny, F. Köckerling, J. Kukleta, K. LeBlanc, D. Lomanto, M. C. Misra, S. Morales-Conde, B. Ramshaw, W. Reinpold, S. Rim, M. Rohr, R. Schrittwieser, Th. Simon, M. Smietanski, B. Stechemesser, M. Timoney, P. Chowbey

**Affiliations:** 1 Hernia Center Rottenburg am Neckar, Winghofer Medicum, Röntgenstr.38, 72108 Rottenburg, Germany; 2 Division of Gastroenterological and General Surgery, Mayo Clinic, 200 First Street SW, Rochester, MN 55905 USA; 3 Department of General, Visceral, Vascular and Pediatric Surgery (Department of Surgery I), University Hospital of Wuerzburg, Oberduerrbacher Strasse 6, 97080 Wuerzburg, Germany; 4 Department of General Surgery, Halifax Health, Daytona Beach, FL USA; 5 Department of Surgery, Lutheran Medical Center, SUNY Health Science Center, Brooklyn, 65 Cromwell Avenue, Staten Island, NY USA; 6 Department of General, Visceral and Oncological Surgery, Wilhelminenspital, 1171 Vienna, Austria; 7 Department of Surgery and Center for Minimally Invasive Surgery, Vivantes Hospital, Neue Bergstr. 6, 13585 Berlin, Germany; 8 General, Visceral, Abdominal Wall Surgery, Klinik Im Park, Grossmuensterplatz 9, 8001 Zürich, Switzerland; 9 Minimally Invasive Surgery Institute and the Fellowship Program, Surgeons Group of Baton Rouge of Our Lady of the Lake Physician Group, Baton Rouge, LA USA; 10 Minimally Invasive Surgical Center, KTP Advanced Surgical Training Center, YYL School of Medicine, National University Hospital, Kent Ridge Wing 2, 5 Lower Kent Ridge Road, Singapore, 119074 Singapore; 11 Division of Minimally Invasive Surgery, J P N Apex Trauma Centre, All India Institute of Medical Sciences, Angari Nagar, New Delhi, 110029 India; 12 Unit of Innovation in Minimally Invasive Surgery, University Hospital “Virgen del Rocío”, Sevilla, Spain; 13 Department of Surgery, Gross-Sand Hospital Hamburg, Gross-Sand 3, 21107 Hamburg, Germany; 14 Department of General Surgery, Katutura State Hospital, PO Box 81233, Olympia, Windhoek, Namibia; 15 Department of Surgery, LKH, Muerzzuschlag, Tragösserstrasse 1 und 1a, 8600 Bruck/Mur, Austria; 16 Department of Surgery, GRN‐Klinik Sinsheim, Weinheim, Germany; 17 Ceynowa Hospital, Wejherowo, Poland; 18 Hernienzentrum Köln, Zeppelinstr. 1, 50667 Köln, Germany; 19 Minimal Access, Metabolic, and Bariatric Surgery, Max Healthcare Institute Ltd., 2 Press Enclave Road, Saket, New Delhi, India

## Section 5: complications

### Management of bowel injury during laparoscopic ventral incisional hernia repair

#### Michael Timoney, Sean Rim, George Ferzli

*Search terms:* “laparoscopic ventral hernia repair” AND “enterotomy” AND “mesh”

A systematic search of the literature was performed in January 2012 using Medline, PubMed, Cochrane library, and reference lists. The search found 27 articles, and 9 were added by hand search. However, only 12 articles were suitable for this review in terms of content.

Key questions

- What are the incidences of bowel injury, and what are the safest techniques for avoiding them?
- What is the safest management for bowel injury, and do alternatives exist?

##### Statements

**Table:** 

Level 1	The incidence of iatrogenic enterotomy during laparoscopic ventral hernia is 1.78 %. The mortality rate for these patients is 2.8 %
	In most cases (92 %), the small bowel is injured
	The most frequent causes are rough adhesiolysis and the use of energized dissection close to the adherent bowel
Level 4	The risk of bowel injury during laparoscopic ventral hernia repair (LVHR) is related to the need for extensive adhesiolysis and to inexperience
	The extent of the bowel injury and contamination dictate the type of repair
	Bowel injury does not always require conversion to open repair
	The LVHR can be delayed for patients who have increased risk factors for the development of mesh infection
	Bowel injury does not preclude immediate LVHR

##### Recommendations

**Table:** 

Grade C	Adhesiolysis should be performed close to the abdominal wall and not near the bowel
	Sharp dissection techniques should be preferred, and the use of energized dissection near the bowel should be avoided
	Conversion to laparotomy is advisable if the surgeon is not proficient with laparoscopic bowel repair techniques
	A primary open repair is advisable in the presence of gross spillage. An open prosthetic repair may be undertaken if conditions remain sterile
	A small laparotomy away from the hernia defect may be used to repair a bowel injury and may be followed by continuation of LVHR
	If a bowel injury is repaired laparoscopically, LVHR may be performed after an observation period of 3–7 days during parenteral antibiotic therapy if no evidence of infection is observed
	An LVHR may be performed in the event of bowel injury repaired immediately with minimal spillage, but this option requires experience with laparoscopic repair of bowel injury

##### Introduction

The first laparoscopic repair of a ventral incisional hernia (LVHR) was reported by LeBlanc and Booth [1] in 1993. Approximately 90,000 ventral incisional hernia repairs are performed in the United States each year. The LVHR procedure continues to gain increasing popularity over open repair. The recurrence rates for LVHR and open repairs are similar. Complications of the laparoscopic technique tend to be fewer but may be more serious, mainly due to a higher incidence of iatrogenic enterotomies [2, 3].

##### Avoiding bowel injury during LVHR

The management of bowel injury during LVHR remains controversial. In a recent review, LeBlanc et al. [4] reported an iatrogenic enterotomy incidence of 1.78 % with LVHR and an overall mortality rate of 2.8 %. In the subset of patients whose injury was missed during the initial operation (18 %), the mortality rate reached 7.7 %. Predictably, the small bowel was injured in 92 % of the reported cases. A recent Cochrane review showed an iatrogenic enterotomy rate of 1.55 % with LVHR versus 0.63 % with the open approach [2–6].

Bowel injuries are classified in one of three categories. Immediately recognized injuries result either from bowel trauma during initial port insertion or from bowel manipulation, especially adhesiolysis. Bowel injuries sustained during adhesiolysis may be missed, to be recognized postoperatively by the development of sepsis during the first 24 h. Delayed injuries occur from progression of a thermal injury caused by energized dissection such as monopolar electrosurgery or ultrasonic dissection. These present within the first 5 days postoperatively [7–9].

Avoiding bowel injury is of utmost importance during LVHR. It is advisable to gain access to the abdominal cavity via an open technique far removed from the hernia or scar. Sharp dissection should always be used in areas of dense adhesions, particularly when the presence of bowel is suspected. Again, the use of energized dissection close to bowel may cause delayed injuries, with significantly increased morbidity and mortality [7].

##### Conversion to laparotomy for bowel injury sustained during LVHR

Management is best dictated by the extent of injury and contamination and by the level of the surgeon’s skill and experience. Options include immediate conversion for open bowel and hernia repair with or without mesh. If the surgeon is adept at laparoscopic bowel repair and contamination is limited, the injury may be repaired laparoscopically and the LVHR performed immediately. An alternative is to repair the bowel and delay the hernia repair until after a period of inpatient observation and administration of parenteral antibiotics [7, 10].

If the surgeon lacks experience with laparoscopic bowel repair, an immediate conversion to a laparotomy is advisable. In such a case, the bowel injury is repaired and the hernia defect managed according to the extent of contamination. In the presence of gross spillage and contamination, the hernia should be repaired primarily without the use of mesh [6, 11].

In 2010, Itani et al. [3] reported a series of 73 patients who underwent conversion to an open technique for bowel injury with minimal contamination during LVHR. In three patients, the enterotomy was repaired, and the herniorrhaphy was performed with polypropylene (PP) mesh laparoscopically. None of the patients who underwent conversion to laparotomy, including those in whom mesh was placed, experienced a surgical-site infection.

##### Alternative methods for dealing with bowel injury during LVHR

In the event of a bowel injury, there are several alternatives to conversion to laparotomy. Carbajo et al. [11] and Heniford et al. [6] both have described a case in which a minilaparotomy was performed to repair the bowel injury. The incision was made away from the hernia and under direct visualization with the laparoscope. The injured bowel was exteriorized through the incision and repaired extracorporeally. The incision then was closed, and the LVHR was resumed.

In the presence of gross contamination, another valid option entails laparoscopic repair of the injury, with postponement of the herniorrhaphy to a later date. Lederman and Ramshaw [5] reported a series of nine patients who sustained an iatrogenic enterotomy during LVHR. After repair of the injury, the patients were observed for an average of 3 days while receiving intravenous antibiotics. With this regimen, seven of the nine patients had successful completion of their LVHR [7]. These authors identified several factors that increased the risk of enterotomy including extensive adhesiolysis taking longer than 3 h, chronic obstruction, inflamed bowel, and mesh incorporation into bowel. The presence of these factors or the recognition of a visceral injury should prompt the surgeon to consider delaying the repair of the hernia until it is certain that the patient shows no signs of intraabdominal infection.

Some authors advocate immediate repair of bowel injuries and completion of the LVHR in the same setting. Carbajo et al. [11] reported eight patients who underwent laparoscopic repair of enterotomies followed by immediate LVHR. Similarly, Heniford et al. [6] reported five patients with hollow organ injuries that were repaired, with the herniorrhaphy completed laparoscopically. The overriding principles in such cases dictate that contamination be minimal or absent and that the surgeon be skilled at laparoscopic repair of bowel.

Finally, the use of biologic mesh also has been described as a safe method for completing LVHR in the presence of contamination. Although synthetic mesh generally is preferred over biologic mesh in terms of recurrence prevention, biologic mesh has been used successfully in contaminated and infected fields.

In 2004, Franklin et al. [12] described their experience with the use of porcine-derived prosthetic mesh in 43 patients who underwent successful LVHR in a contaminated field. Details of the contamination are vague but included bowel resection, strangulation, and prior mesh infection. One patient experienced a wound infection and a fistula. The authors report no recurrences.

### Unrecognized enterotomy

#### Karl A. LeBlanc, Matthias Rohr

*Search terms:* “open abdomen” AND “enterotomy”; “damage control laparotomy” AND “enterotomy”; “laparoscopy” AND enterotomy”; “enterotomy” AND “avoidance”; “inadvertent enterotomy” AND “hernia repair”; “enterotomy” AND “hernia repair”; “enterotomy” AND “hernia repair” AND “peritoneal contamination”

The search was limited to English literature and non-English literature with an English abstract. The search was based on Pub Med and Embase databases as well as on the Cochrane register using the aforementioned search terms from 1960 to 2011. A total of 174 articles met the search criteria, but only 78 of these articles adequately dealt with the subject matter. Of these, 32 qualified with respect to levels of evidence.

##### Statements

**Table:** 

Level 2A	Reoperation will be necessary
	The recommended method of repair or resection of the intestinal injury cannot be supported
	Mesh explantation with primary repair of the hernia is recommended
Level 4	Evidence supports a laparotomy but not the specific treatment of the intestinal injury
	• Repair or resection both are appropriate
	• Mesh explantation is necessary
	• Primary repair of the hernia is recommended
Level 5	When this is suspected, repeat laparoscopy or laparotomy is necessary
	• Repair or resection each is appropriate
	• Mesh explantation is necessary
	• Primary hernia repair is recommended

##### Recommendations

**Table:** 

Grade B	Surgeons use either open or laparoscopic procedure to re-explore the patient if there is a suspicion of a missed iatrogenic enterotomy or
	to repair the injury, resect the injured segment, or create a stoma depending on the injured organ and the clinical situation
Grade C	Mesh explantation should be performed
	Primary repair of the hernia, if feasible, is with current evidence deemed to be the option

##### Introduction

The first report describing repair of incisional and ventral hernias by the laparoscopic method did not usher in a rapid adoption as did laparoscopic cholecystectomy a few years earlier [1]. Since the initial report, subsequent reports have confirmed the efficacy of laparoscopic repair. Despite its success, one of the most feared complications is an unrecognized iatrogenic enterotomy. The risk of this complication existed with open repair, but its recognition during laparoscopic repair is more difficult, and the injury may be missed. This is further compounded by the earlier discharge of the patient.

The overall incidence of iatrogenic enterotomy during LVHR ranges from 1.78 to 6 % [4, 13, 14]. The reported rate for unrecognized enterotomy ranges from 0.68 to 2.9 % [4, 13, 15]. The rate for unrecognized enterotomy does not differ significantly between open and laparoscopic repairs [2, 4, 16, 17]. The rate of mortality from these iatrogenic injuries within any series ranges from 0.05 to 3.4 % [4, 13–15]. However, among patients in whom the enterotomy is missed, the mortality rises significantly and ranges from 7.7 to 100 % [4, 13–15].

Enterotomy is not completely unavoidable in either the open or the laparoscopic repair, and the consequences of this complication are always serious.

##### Discussion

This systematic review identified a total of 174 articles that met the search criteria, but only 78 adequately dealt with the subject matter. Of these, only 32 qualified for evidence-based surgical recommendations. Collectively, these publications contained very little specific information on unrecognized (missed) enterotomy or its management during reoperation, including management of the hernia itself.

Two level 1A publications evaluated the laparoscopic repair of incisional and ventral hernias but did not specifically deal with the subject of missed enterotomy. Both concluded that a higher rate of injury to an intraabdominal organ appeared to associated with the laparoscopic approach, but this difference was not statistically significant [2, 16].

We found only two papers that discussed the method for repair of the intestinal injury, and only one of which discussed the management of the hernia defect at level 2A. The one paper concluded that for unrecognized enterotomy, “reoperation with closure/resection of the injury in conjunction with mesh explantation is necessary” [18]. The other paper reported that no one method of repair was superior and concluded that either suture or staple closure could be used for primary repair of the injury with equal success [4]. However, it should be noted that, depending on the nature of the injury, including the viscus involved, the clinical condition of the patient, and the reoperative findings, some patients will require creation of an ostomy as part of the treatment. This is supported by the single level 2B study in which the stoma was closed and the hernia repaired 3 months later [19]. The level 4 evidence is summarized in (Table 1) Binenbaum and Goldfarb [23].

**Table:** 

Series	Incidence (%)	Laparoscopy/laparotomy	Primary repair of intestine/resection	Mesh explant/primary hernia repair
Baccari et al. [20]	1	Yes/yes	Resection	Explant/primary repair
Ben-Haim et al. [21]	2	No/yes	Primary repair	Explant/hernia not repaired
Berger et al. [22]	1.3	No/yes	Repair (*n* = 1) Resection (*n* = 1)	Explant/primary repair
Binenbaum and Goldfarb [23]	0.3	No/yes	Resection	Not described
Heniford et al. [6]	1.7	No/yes	Resection	Primary repair
Koehler and Voeller [14]	2.9	No/yes	Resection	Explant/primary repair
Moreno-Egea et al. [13]	1.1	Not mentioned	Not discussed	Not discussed
Perrone et al. [24]	1.6	No/yes	Resection	Not mentioned/primary hernia repair
Wara and Anderson [25]	1.4	Not mentioned	Not discussed	Not discussed
Wright et al. [15]	0.68	No/yes	Not discussed	Explant/not discussed

Baccari et al. [20] are the only authors who perform a laparoscopic examination of the abdomen to ascertain the presence of an iatrogenic enterotomy, and if such an injury is discovered, perform a formal laparotomy and open repair. In the remaining publications, mesh explantation, intestinal resection, and primary repair of the hernia constitute the preferred management of the unrecognized injuries.

Despite the numerous reports on laparoscopic repair of incisional hernias, very few (in fact, only 2) have addressed this problem. As LeBlanc [10] advocated, “a laparotomy will generally be required with bowel resection and explant of the mesh.” Serala [26] recommended that “if there is a high index of suspicion of a missed enterotomy, a planned re-laparoscopy after 24–48 h…should be done.” However, this report does not provide any specific recommendations for the management of either the intestinal injury or the hernia.

##### Conclusions

Based on the relative paucity of high-level data on the management of this serious problem, it seems that the safest approach is repair, resection of the injury, mesh explantation, and primary repair of the fascial defect. If this is not possible laparoscopically or if the clinical condition warrants, open surgical treatment is essential.

### Risk factors for infection in laparoscopic incisional ventral hernia repair

#### P. Chowbey

*Search terms:* “risk factors for SSI,” “risk factors for infection,” “causes of mesh infection,” “laparoscopic ventral/incisional hernia repair,” “perioperative risk factor for infection”

The recommendations relating to the risk factors for infection in laparoscopic ventral/incisional hernia are based on a systematic search and review of the literature performed in Pubmed, Medline, Cochrane Library, EMBASE, the *British Journal of Surgery* database, UK Pubmed Central, Google, Google scholar, Scirus, Ovid, and Directory of Open Journal Access (DOAJ). Of the 38 publications found that covered the topic, 15 statements were considered useful for this research.

##### Statements

**Table:** 

Level 1	Preoperative transfusion may increase the risk of surgical-site infection (SSI)
	Laparoscopic operations lead to a lower incidence of SSI than open operations because the total length of the incisions is shorter, reducing the risk of bacteria entering the subcutaneous space
Level 2	In elderly patients, chronic obstructive pulmonary disease (COPD) and low preoperative serum albumin are independent predictors of wound infections; coronary artery disease (CAD), COPD, low preoperative serum albumin, and steroid use are independent predictors of a longer hospital stay
	Patients who undergo ventral hernia repair with a simultaneous bowel resection show a higher incidence of infectious and noninfectious complications with mesh use
	Wound infection is lower in laparoscopic hernia repair than in open repair due to the decreased extent of tissue dissection
	Mesh, wherever possible, should not be brought in contact with skin to avoid contamination by skin flora. Polyester meshes are associated with the highest incidence of infection, fistualization, and recurrence
	Patients given a prophylactic antibiotic have a lower incidence of SSI
Level 3	Patient operation time is the only significant risk factor associated with infection of mesh graft after incisional hernia repair
	Patient age, American Society of Anesthesiology (ASA) score, smoking, surgery duration, and an emergency setting of the operation are associated with the development of synthetic mesh infection
	Complications are significantly associated with larger hernias, previous herniorrhaphy, longer operating times, and extended hospital stays
Level 4	Patient characteristics that increase the risk of SSI include steroid use, smoking, old age, and underlying disorders such as obesity, diabetes, malnutrition, and remote-site infection
	The source of SSI is skin flora or bacterial contamination from a viscus
	The use of the mesh does not increase the incidence of SSI, although the consequences of the mesh infection may be severe
	If the mesh is placed subcutaneously, SSI is more common than if it is placed in a subaponeurotic premuscular, pre-aponeurotic retromuscular, or preperitoneal space. If infection is present, repair by tension-free nonabsorbable prosthetic implants is not recommended
	A prolonged preoperative hospital stay and preoperative nares colonization with *Staphylococcus aureus* increase the risk of SSI
	The presence of drainage and its duration increases the incidence of SSI. If an indication for drainage exists, it should be as short as possible

##### Recommendations

**Table:** 

Grade A	Laparoscopic repair is associated with a lower risk of SSI and thus is preferred over the open approach
	Before surgery, known risk factors for SSI must be treated if possible
	The operation time and hospital stay must be as short as possible
Grade B	Smoking cessation, glycemic control, and treatment of remote infections should be done before surgery
	Prosthetic mesh insertion with simultaneous bowel resection should be avoided.
Grade C	Preoperative clipping of hair is recommended
	Weight loss before the operation may be considered

##### Discussion

The presence of SSI significantly increases morbidity and mortality [27]. The reported incidence of infection is 10 % for open procedures and 1.1 % for laparoscopic procedures [28]. Laparoscopic procedures lower the risk of infection by reducing wound size, hospital stay, operative time, and the probability of bacteria entering the subcutaneous space [29–32].

The pathogens that frequently cause SSI are *Staphylococcus aureus, Enterococcus* species, and *Escherichia coli,* which usually are sourced from patient’s skin, mucous membranes, or bowel, and rarely from another infected site in the body [32, 33]. The risk factors for infection can be divided into patient- and surgery-related factors.

*Patient-related risk factors.* Gender and SSI are not correlated, but wound infection in 15- to 24-year-old patients averages 10 % and increases significantly in patients older than 65 years [34]. Dunne et al. [35] reported CAD, COPD, and low preoperative serum albumin as independent predictors for infection in elderly patients. Smokers and patients receiving immunosuppressants and steroids also have a greater risk of contracting infection. The risk of infection increases fivefold for smokers and by 9 % for patients receiving steroids [34]. Diabetes and malnutrition also are significant risk factors for infection [36]. Obesity decreases the blood circulation in fat tissue and increases the risk of infection [37]. Other factors such as history of infection, high ASA grades, hypoxia, hypothermia, radiation, and peripheral vascular disease also contribute to an increased risk of SSI [38–41].

*Surgery-related risk factors.* The preoperative factors increasing the risk of infection include shaving of the surgical site, short duration of scrubbing, antiseptic use, and blood transfusion. The SSI rate was 5.6 % for patients who had hair removed by razor compared with 0.6 % for patients who either had their hair removed by depilatory agents or had no hair removal [42]. Blood transfusion increases the risk twofold [43]. Long operating time also predisposes to the risk of infection. Procedures longer than 3–4 h increase the risk [38].

In addition, mesh infection is a major factor contributing to infection. The reported incidence of infection after laparoscopic repairs is 0–3.6 % [44]. A mesh infection rate as low as 0.78 % after laparoscopic repair was reported in a systematic review by Carlson et al. [18]. Polyester meshes and meshes positioned subcutaneously are associated with a high incidence of infection [44, 45]. The use of prosthetic mesh with bowel resection or injury increases the risk of infection many-fold [46]. Also, blood loss during the surgery is a significant risk factor. Postsurgery complications such as seroma, thromboembolism, pulmonary embolism, post-procedure pneumonia, and anemia make the patient more susceptible to infection [47].

To prevent infection, management of these risk factors is important. The risk factors that can be modified should be addressed and managed by adherence to established guidelines and protocols [48]. Cessation of smoking before the surgery reduces the risk of postoperative SSI in addition to other cardiovascular and respiratory benefits. No data are reported on the effect of preoperative parenteral or enteral nutrition on the incidence of SSI [49]. Strict preoperative glycemic control with maintenance of intraoperative normothermia is necessary [50]. Remote infection, especially when mesh is being implanted, should be treated and resolved completely before the surgery. Preoperative hair removal should be avoided, and clipping should be performed instead [42]. Prophylaxis with antibiotics administered half an hour before surgery produces the best results [51]. During surgery, careful attention to proper surgical technique and timely completion of the operation also reduce the risk of SSI.

### Mesh infection

#### F. Köckerling, P. Chowbey, M. C. Misra

*Search terms*: “incisional hernia,” “ventral hernia,” “laparoscopic incisional hernia repair,” “laparoscopic ventral hernia repair,” “hernia repair and mesh infection,” “mesh infection,” “hernia repair and wound infection,” “laparoscopic ventral hernia repair and mesh infection,” “incisional hernia repair and mesh infection”

A systematic search of the available literature was performed in July 2012 based on Medline, PubMed, and the Cochrane Library, as well as relevant journals and reference lists using the aforementioned search terms. The first search found 118 relevant articles. In a second-level search, four articles were added. A total of 15 publications were used for this systematic review.

Key questions

- How should a mesh infection be treated?
- When a mesh has to be removed?
- Should the hernia defect be closed?
- Does implantation of biologic meshes play any significant role?
- When is it safe to re‐implant a synthetic mesh after an infection had occurred?
- How long should be waited for the reoperation?
- In which cases is a vacuum‐assisted therapy indicated?

##### Statements

**Table:** 

Level 1A	The rate of mesh infections after laparoscopic ventral and incisional hernia repair is low (1 %)
	The mesh does not need to be removed in all cases of wound infection after laparoscopic ventral and incisional hernia repair
Level 3	Infected expanded polytetrafluorethylene (ePTFE) meshes require removal significantly more often than PP-based meshes
Level 5	Case reports in the literature indicate that salvage of infected meshes after laparoscopic ventral and incisional hernia repair is possible
	Conservative management of mesh infection after laparoscopic ventral and incisional hernia repair can be attempted by percutaneous drainage, drain irrigation with gentamycin 80 mg in 20 ml of saline 3 times a day, and intravenous antibiotics
	When the conservative treatment of a mesh infection after laparoscopic ventral and incisional hernia repair fails, all the same options as for mesh infection after open repair need to be considered depending on the individual circumstances of the patient
	The following options may be used in the treatment of mesh infections after open repair:
	Mesh removal and primary skin closure, with repair of the defect repeated after 6–9 months.
	Mesh removal using the component separation technique and vacuum-assisted closure or open-wound dressing
	Mesh removal, repair with biologic mesh, and vacuum-assisted closure or open-wound dressing
	Mesh salvage and vacuum-assisted closure or open-wound dressing

##### Recommendations

**Table:** 

Grade B	An infected ePTFE mesh after laparoscopic ventral and incisional hernia repair should be removed
Grade D	Preservation of an infected composite mesh after laparoscopic ventral and incisional hernia can be attempted by either interventional or conservative treatment using percutaneous drainage, drain irrigation with gentamycin, and intravenous antibiotics
	If the conservative treatment fails or is not justified for any reason, the established options for treatment of mesh infections after open repair should be used
	Because only the options for individual cases are reported, a decision must always be made in accordance with the findings for the individual patient

An important advantage of the laparoscopic intraperitoneal onlay mesh (IPOM) technique over open repair of incisional and ventral hernias is the lower rate of wound and mesh infections. One metaanalysis demonstrated that laparoscopic repair of incisional and ventral hernias significantly is attended by fewer wound infections and less need for mesh removal (level 1A) [16].

In the metaanalysis by Sauerland et al. [2], the local infection rate in the laparoscopic group was 3.1 versus 13.4 % in the open group (*p* < 0.00001). A local infection requiring mesh removal was found in 0.7 % of the laparoscopic group and 3.5 % of the open group (*p* = 0.09). This trend also was seen for infections resulting in mesh removal. In this metaanalysis, the rate of wound infections after laparoscopic repair was 2.23 %.

Out of these 2.3 % wound infection did not lead to mesh removal in two third of cases, but one third of wound infections did result in mesh removal [16]. In a pooled data analysis (level 2A) by Pierce et al. [52], wound infections were found in 1.3 % of cases after laparoscopic repair and mesh infections in 0.9 % of the cases, whereas after open operation, the wound infection rate was 10.9 %, and the mesh infection rate was 3.2 % (*p* < 0.0001). In a large clinical case series and case analyses (level 3), mesh infections were detected after laparoscopic IPOM in 0.78 % (*n* = 6,206) [18], 0.90 % (*n* = 4.582) [52], and 0.70 % (*n* = 850) [6] of the patients. In the literature, case reports on the treatment of mesh infections after laparoscopic repair of incisional and ventral hernias discuss both mesh removal [24, 53] and mesh salvage [54, 55].

For interventional and conservative treatment of a mesh infection after laparoscopic repair of incisional and ventral hernias, Aguila et al. [54] and Trunzo et al. [55] advocate percutaneous drainage of accumulated pus around the mesh and insertion of a drain through which irrigation with gentamycin 80 mg in 20 ml saline solution is carried out three times daily together with intravenous antibiotic treatment.

Treatment of mesh infection also depends on the material used. In a comparative study (level 2B), Hawn et al. [56] demonstrated significantly less need to remove a PP mesh than a PTFE mesh because of a mesh infection (*p* < 0.0001). Petersen et al. [57] also showed that for mesh repair of incisional hernias, with which mesh infection occurring in 8.1 % of cases after the use of ePTFE and in 3.9 % after the use of PP, in no case was it possible to salvage the infected ePTFE mesh, whereas all the infected PP meshes were preserved. Hence the chances of mesh salvage after infection are greater with PP meshes than with ePTFE meshes, which usually have to be explanted.

If an interventional conservative attempt at treating a mesh infection after laparoscopic IPOM proves unsuccessful or if from the outset the circumstances no longer allow preservation of the mesh, various options can be used for mesh infections after mesh repair of incisional and ventral hernias [58–61], including

- Mesh removal and primary skin closure, with the repair repeated after 6–9 months.
- Mesh removal using the component separation technique, with the skin left open and vacuum-assisted wound closure or open-wound dressing applied.
- Mesh removal, repair of the defect with a biologic mesh, leaving the skin open and applying vacuum-assisted wound closure or open-wound dressing.
- Mesh salvage, with the skin left open, and vacuum-assisted wound closure or open-wound dressing applied.

Because the treatment options available in the literature relate only to individual cases or to small case series, currently, no concrete evidence-based recommendation can be made for the optimal management that gives the best results. Instead, the surgeon must decide in the individual case which option is best for the individual patient. There is an absolute need for further studies.

### Postoperative seroma: risk factors, prevention, and best treatment

#### J. Bingener, M. Rohr

*Search terms:* “hernia” AND “ventral and laparoscopy” AND “laparoscopic surgery and seroma” AND “incisional hernia and abdominal wall hernia and laparoscopy/or laparoscopic surgery/or hernioplasty”

The search resulted in a total of 946 citations from Ovid Medline for the period 1948–August 2011, PubMed including prepublication, Embase for the period 1988 to the 33rd week of 2011, evidence-based medicine reviews and the Cochrane Register, and the Web of Science for the period 1993–2011.

The search produced 27 studies (2 prospective and 25 retrospective studies) [6, 16, 21, 62–85].

### Incidence

#### Statements

**Table:** 

Level 4	Seroma can be detected by ultrasound in up to 100 % of patients
Level 4	Seroma formation peaks at about postoperative day 7
Level 4	Seroma resolution is almost complete at 90 days
Level 2B	Up to 30 % of patients who experience development of seroma become symptomatic

##### Recommendations

**Table:** 

Grade B	Patients should be informed on the possibility of both asymptomatic and symptomatic seroma formation

The reported incidence of seroma after laparoscopic ventral hernia repair varies widely from 3 to 100 %, with a peak presentation at 7 days postoperatively and almost complete resolution by 90 days after surgery [16, 62, 65, 66, 68, 83]. Whether all seromas constitute a complication or represent only an inconsequential epiphenomenon of laparoscopic ventral incisional hernia repair is unclear. According to the current surgical literature, up to 35 % of patients will become symptomatic, with pain, pressure, or erythema [66]. In some cases, chronic seroma will develop. Most of the studies reviewed for these Guidelines did not distinguish between clinically significant and asymptomatic seromas.

Whereas clinical retrospective studies often report the incidence of seroma to be 4–78 % [6, 74, 77, 84], a prospective study with close and ongoing ultrasound follow-up assessment reported a 100 % incidence of seroma at 7 days, with all but complete spontaneous resolution by 90 days [66]. The study used mesh, tacks, and sutures. Up to 30 % of patients become symptomatic from the seroma [71].

### Risk factors

#### Statements

**Table:** 

Level 2B	Laparoscopic and open repairs are compared (trials with opposing results)
Level 2B	Nonreducible hernia is a risk factor
Level 3	Seroma may be more common with IPOM than with transabdominal preperitoneal PP (TAPP) LVHR
Level 2B	The incidence increases with the number of prior abdominal incisions
Level 2B	The hospital center (within the VA system) is an independent predictor of seroma
Level 5	Sutures through the hernia sac predispose to sustained seroma

A large VA study identified the following risk factors for seroma in open and laparoscopic hernia repairs: an irreducible hernia, an increased number of prior abdominal incisions, and a hospital center within the VA system (as an independent predictor of seroma) [62]. The finding that hospital centers are linked to seroma formation suggests that intraoperative technical factors may play a role. The transabdominal preperitoneal repair for primary ventral and umbilical hernias may decrease the likelihood of seroma formation [68]. Randomized trials yield conflicting results regarding the likelihood of seroma formation with laparoscopic or open repair [16, 64].

### Prevention

#### Statements

**Table:** 

Level 2B	Cauterizing of the hernia sac may lead to less seroma formation
Level 2B	Placement of a quilting stitch does not affect seroma formation
Level 2B	Double-crown stapling does not decrease seroma formation
Level 4	No specific mesh type is related to seroma formation
Level 4	Compression dressing for 1 week reduces the occurrence of seroma

##### Recommendations

**Table:** 

Grade C	Surgeons can attempt cauterization of the hernia sac to prevent seroma formation
Grade C	Surgeons may place a pressure dressing in an attempt to reduce the incidence of seroma

To prevent seroma, a small randomized study (JADAD score: 0) determined that if the hernia sac was cauterized by electrocautery or ultrasonic energy, the seroma frequency was decreased from 25 to 4 % [85]. Other similar trials have reported that placing a quilting stitch or double-crown stapling to decrease the dead space did not affect seroma formation [67]. Studies suffer from small numbers. One study reported that the placement of a compression dressing for 1 week reduced the occurrence of seroma [82].

### Treatment

#### Statements

**Table:** 

Level 2B	The majority of seromas resolve spontaneously
Level 4	Aspiration is often effective
Level 4	Repeated aspiration may lead to mesh infection.
Level 5	An abdominal binder does not reduce seroma formation (unpublished randomized controlled trial [RCT] data)
Level 2B	The length of abdominal binder use does not affect seroma formation

##### Recommendations

**Table:** 

Grade B	The majority of seromas should be expected to resolve spontaneously
Grade B	Patients should be informed about the risk of infection if a seroma is repeatedly aspirated

The recommendations are strongest for informing patients about the possible occurrence of seromas and the expectation that the majority will resolve spontaneously [62, 63, 66, 83]. Given the clinically important consequences of mesh infection as a possible complication of repeated seroma aspiration, this recommendation also may be considered stronger (level B), although it is based on only level 4 evidence [62, 74].

The importance of applying a pressure dressing was supported by one study with methodologic limitations [82] and may be contradicted by the findings regarding binder placement, a circumferential pressure dressing.

### Postoperative bulging

#### M. Rohr

*Search terms:* “laparoscopic hernia repair” AND “LVHR” AND “incisional hernia” AND “ventral hernia” AND “postoperative bulging abdominal wall” AND “abdominal wall bulging” AND “abdominal wall hernia and bulging” AND “complication bulging” AND “incisional hernia and bulging” AND “bulging after hernia repair” AND “long term results”

A systemic search of the available literature was performed in August 2011 based on Medline, PubMed, the Cochrane Library, and relevant journals and reference lists using the aforementioned search terms. Of the 54 articles found, only four dealt with “bulging” after laparoscopic hernia repair.

Key questions

- Is it a real problem?
- Is it avoidable?

##### Statements

**Table:** 

Level 2B	Abdominal bulging is a specific problem associated with laparoscopic repair of large incisional hernias
	In 1.6–17.4 % of patients, bulging is observed after laparoscopic ventral/incisional hernia repair
	Symptomatic bulging is rare
Level 2C	Symptomatic bulging, although not a recurrence, is an important negative outcome of laparoscopic ventral hernia repair
Level 4	Hernia defect closure eliminates postoperative seroma and consequently bulging

##### Recommendations

**Table:** 

Grade B	Symptomatic bulging, although not a recurrence, requires a new repair
Grade B	In asymptomatic patients, “watchful waiting” seems justified
Grade C	The addition of defect closure eliminates postoperative seroma and consequently bulging

##### Introduction

Besides pain, patients sometimes report postoperative abdominal bulging, which can be cosmetically dissatisfying. The anatomic basis for this problem lies in the fact that neither the hernia orifice nor the rectus diastasis (if present) was closed during laparoscopic hernia repair. These issues, relevant mainly with large hernias, should be discussed with the patient preoperatively [2].

##### Bulging: is it a real problem and avoidable?

This section concerns the prevalence, diagnosis, clinical significance, and treatment of bulging in the area of laparoscopic repair of ventral hernia caused by mesh protrusion through the hernia opening, but with intact peripheral fixation of the mesh forming an adequate repair [86].

In a study of 765 patients who underwent laparoscopic ventral hernia repair, all the patients with swelling in the repaired area (*n* = 29) were identified and subjected to further examination by computed tomography (CT). The exam showed that 17 patients (2.2 %) had a recurrence hernia. For an additional 12 patients (1.6 %), the CT indicated only bulging of the mesh but no recurrence. Bulging was associated with pain in four patients, who underwent relaparoscopy and got a new, larger mesh tightly stretched over the entire previous repair. Eight asymptomatic patients agreed to “watchful waiting.” All the patients remained symptom free during a median follow-up period of 22 months.

Symptomatic bulging requires a new repair and must be considered as an important negative outcome of laparoscopic ventral hernia repair. In asymptomatic patients, “watchful waiting” seems justified [86].

In the prospective study of Kurmann et al. [87], the long-term results after laparoscopic repair of large incisional hernias remain to be determined. The study was designed to compare early and late complications between laparoscopic and open repairs in patients with large incisional hernias. In this study, 56 patients with a hernia diameter of 5 cm or larger who underwent open incisional hernia repair were compared with 69 patients who underwent laparoscopic repair. The median follow-up period was 32.5 months (range 1–62 months) in the laparoscopic group versus 65 months (range 1–80 months) in the open group [3]. The recurrence rate did not differ between the two techniques, but abdominal bulging was identified as a specific problem associated with laparoscopic repair of large incisional hernias (17.4 %) because it was rare (7.1 %) after open repair [87].

To reduce the incidence of seromas or bulging, Orenstein et al. [88] modified their LVHR approach to routine closure of the transabdominal defect (“shoelacing” technique) before mesh placement. In their study, 47 consecutive patients undergoing LVHR with shoelacing were reviewed retrospectively. The LVHR technique with defect closure confers a strong advantage in hernia repair, shifting the paradigm toward more physiologic abdominal wall reconstruction.

Orenstein et al. [88] reported this approach to be safe and comparable with historic control studies. While providing reliable hernia repair, the addition of defect closure in their patients essentially eliminated postoperative seroma, and routine use of the shoelace technique reduced bulging. Both procedures are thus advocated in ventral hernia repair.

##### Comment

Symptomatic bulging, although not a recurrence, requires a new repair and must be considered as an important negative outcome of laparoscopic ventral hernia repair. For asymptomatic patients, “watchful waiting” seems justified.

Abdominal bulging is a specific problem associated with laparoscopic repair of large incisional hernias. It occurs in 2–20 % of patients. This wide range may be attributable in part to interpretation by the examiner and the opinion of the patient, but evidence for this is limited. There is an urgent need for more studies regarding this topic.

### Chronic pain: risk factors, prevention and treatment

#### J. Bingener, W. Reinpold, P. Chowbey

*Search terms:* “hernia” AND “ventral laparoscopy” AND “laparoscopic surgery” AND “postoperative complications or recurrence or pain” AND “postoperative or surgical wound infection” AND “prosthesis design/failure/implantation/device removal” AND “pain”

The search resulted in a total of 946 citations from Ovid milliner for the period 1948–August 2011, PubMed including prepublication, Embase for the period 1988 to the 33rd week of 2011, evidence-based medicine reviews and the Cochrane Register, and the Web of Science for the period 1993–2011.

Chronic pain after laparoscopic ventral hernia repair has been addressed by 3 metaanalysis/systematic reviews, 13 RCTs, 5 comparative-cohort studies, and 19 single-cohort studies [2, 3, 17, 19, 52, 74, 76, 79, 81, 82, 92–121] The randomized trials were of fair to poor quality, which influenced the levels of evidence assigned to the statements and recommendations. From this review, the following statements and recommendations are made.

### Risk factors

#### Statements

**Table:** 

Level 2A	The LVHR technique results in chronic pain for 2–4 % of patients
Level 2C	Recurrence is associated with chronic pain (open and laparoscopic)
Level 3	Non-midline laparoscopic ventral hernia repair is more often associated with chronic pain
Level 4	The LVHR technique may lead to residual pain in up to 26 % of patients.
Level 2B	Acute postoperative pain (non-procedure-specific) is experienced

### Non-procedure-specific risk factors

#### Statements

**Table:** 

Level 2B	Age
Level 2B	Gender
Level 2B	Preoperative pain
Level 2B	Psychosocial factors
Level 2B	Cognitive distortion

### Prevention

#### Statements

**Table:** 

Level 2B	Local anesthetic at suture sites during surgery significantly decreases acute early pain.
Level 2B	Pain pump placement makes no difference in acute or chronic pain
Level 4	Tissue glue results in “low levels of postoperative pain.”
Level 2B	The visual analog scale (VAS) shows no difference between absorbable and permanent fixation sutures at 3 months, but quality-of-life (QOL) differences (physical activity) are experienced
Level 2B	Pain is not correlated with the number of tacks
Level 3	No consistent difference between PP and other LW meshes is shown by pain scores
Level 4	Absorbable fixation tacks are associated with few cases of chronic pain at 1 year
Level 2A	Transfascial sutures with tacks do not result in higher pain scores than tacks only
Level 2B	Permanent suture fixation at 2- to 3-cm intervals results in a higher number of patients with pain 6 months postoperatively compared with tacks-only fixation
Level 2B	Pain frequency after permanent suture fixation at 6 months is similar to that for tacks-only fixation
Level 2B	A permanent corner suture plus double-crown tacks results in higher VAS scores than permanent sutures only in hernias with a defect size <5 cm.

##### Recommendations

**Table:** 

Grade B	Patients should be informed that laparoscopic ventral hernia repair may lead to prolonged pain
Grade B	Surgeons should strive to limit acute pain as a risk factor for chronic pain
Grade B	Surgeons should use intraoperative suture-site injection of local anesthetic
Grade D	The evidence is inconclusive whether the type of suture, tacks, glue, or mesh alters the incidence of chronic pain

### Treatment

#### Statements

**Table:** 

Level 2B	The lidocaine patch does not significantly reduce postoperative acute or chronic pain
Level 4	Local injection after surgery at suture sites can resolve pain
Level 4	Suture removal can resolve chronic pain
Level 4	Mesh removal can resolve chronic pain
Level 4	Multimodality pain treatment can resolve chronic pain

##### Recommendations

**Table:** 

Grade C	Injection of local anesthetic at suture sites can be considered in the treatment of chronic pain
Grade C	Removal of suture, tacks, or mesh can be considered in the treatment of chronic pain
Grade C	Multimodality pain treatment may be necessary in the treatment of chronic pain

##### Introduction

It is well established that surgical injury can lead to chronic pain, defined by the International Association for the Study of Pain (IASP) as pain lasting for 3 months or more [89]. The components and risk factors for postoperative pain can be classified as patient factors, intraoperative factors (tissue damage, mesh type, type of anesthesia), and postoperative factors (type of analgesia. Patient factors [90, 91] contribute to postoperative pain perception but were not investigated in the studies available for review.

The studies on this topic included in the review showed substantial heterogeneity, with varying definitions of pain. The definition of chronic/prolonged pain was often vague, ranging from longer than 24 h to longer than 6 months. Furthermore, the trial designs and reporting were not uniform, further limiting the comparability of the outcomes. This also was noted in the reported metaanalyses of laparoscopic ventral hernia repair [2, 17, 92].

Specific studies examining chronic pain in patients with ventral hernia repair are infrequent. We may be able to extrapolate some findings from other studies relevant to the assessment of pain syndromes and chronic pain. In inguinal hernia repair, e. g., other preoperative chronic pain conditions not related to the groin [122–124] as well as severe early postoperative pain [124–128] after groin hernia repair are significant risk factors for chronic pain. Ventral hernia recurrence was reported as a risk factor for chronic pain in a large Veteran’s Affairs Medical Centers survey [94]. A nonmidline (e.g. lumbar) location is accompanied by both more pre- and postoperative pain [97].

Attempts at prevention of chronic pain after laparoscopic ventral hernia repair have involved mainly different mesh fixation techniques. Unfortunately, the results of these studies often are contradictory [93, 96, 98, 101, 102, 118–121]. Local anesthetic infiltration is reported to be of benefit [103, 104]. However, one report on infusion of local anesthetic showed no benefit [95]. Regrettably, the treatment of chronic pain after laparoscopic ventral hernia repair is described in small case series that address local and systemic pain treatment as well as removal of fixation or mesh components or excision of neuroma [102, 120].

### Recurrence after laparoscopic ventral and incisional hernia repair: risk factors, mechanism, and prevention

#### P. Chowbey

*Search terms:* “incisional hernia” and “recurrence”, “recurrence” and “risk factors” and “incisional hernia”, “incisional hernia” and “prevention of recurrence”, “incisional hernia” and “mechanisms of recurrence”

A systematic search of the literature was performed using Pubmed, Medline, the Cochrane Library, EMBASE, the *British Journal of Surgery* database, Google scholar, Scirus, Ovid, and the Directory of Open Journal Access (DOAJ). The search found 34 publications that covered the topic, 19 of which were useful for this systematic review.

### Risk factors for recurrence

#### Statements

**Table:** 

Level 1	The existing literature does not document the superiority of any one mesh fixation technique in relation to recurrence
Level 3	Size of the hernia (≥10 cm), body mass index (BMI) (≥30 kg/m^2^), history of previous open repair or failed hernia repair, and perioperative complications including SSI are risk factors for hernia recurrence irrespective of the technique
Level 3	The risk factors for recurrence include patient status, underlying disease, and perioperative factors (i.e., surgical techniques, postoperative complications, deep abscesses, and early reoperations)
Level 3	Smokers with previous failed repair attempts have a higher risk of recurrence
Level 3	Postoperative mesh infection requiring removal of mesh is a predictor of recurrence
Level 3	Higher incidence of seroma formation and recurrence are reported in cases managed with dual mesh
Level 3	Repetition of a previously inadequate technique for recurrent hernia usually fails

##### Recommendations

**Table:** 

Grade B	Risk factors predisposing to recurrence after laproscopic ventral or incisional hernia repair should be eliminated before surgery as far as possible
Grade B	Insufficient incision scar coverage with mesh, SSIs, and gastrointestinal complications should be avoided

### Mechanisms of recurrence

#### Statements

**Table:** 

Level 3	The mechanism for recurrence of ventral hernia described in the literature in decreasing order of frequency are infection, lateral detachment of the mesh, inadequate mesh fixation, inadequate mesh, inadequate overlap, missed hernias, raised intraabdominal pressure, and trauma
Level 4	The mechanism of recurrence can be improperly placed transfascial sutures, overly large bites of mesh causing excessive tension, and, ultimately, a hole in the mesh.
Level 4	Mesh shift may be a precursor to hernia recurrence. Mesh tends to shift away from the operative side, leading to recurrence. Recurrence may be a two-step process, beginning first with intraoperative mesh shift followed by additional factors (e.g. mesh contraction) that may accentuate the shift and lead to recurrence
Level 4	Recurrence can occur at defects occurring at transfascial suture sites of previous laparoscopic ventral hernia mesh repair

##### Recommendations

**Table:** 

Grade B	A strictly standardized technique to avoid failures such as mesh overlap less than 3 cm, improper fixation, and mesh contraction and invagination into the hernial defect should be used
Grade C	Optimal preoperative treatment for patients with increased intraabdominal pressure in conditions such as COPD, chronic cough, and obesity should be considered

### Prevention of recurrence

#### Statements

**Table:** 

Level 1	Recurrences can be prevented by using increased overlap of the biomaterial and dual methods of fixation (tacks and transfascial sutures)
Level 3	Incisional and ventral hernias larger than 2 cm are preferably repaired using a prosthesis because primary repair has a high rate of recurrence
Level 3	Use of mesh in a repair of incisional hernia reduces the risk of recurrence
Level 3	A mesh overlap of at least 5 cm and fixation of the lower margin of the mesh under direct vision to Cooper’s ligaments appear to confer increased strength and durability and contribute to low hernia recurrence rates in patients with suprapubic hernias
Level 4	Meticulous use of transfascial sutures with other fixation methods improves recurrence rates for high-risk obese patients
Level 4	Insufficient coverage of the incision scar is a risk factor for recurrence after laparoscopic repair of ventral and incisional hernia; hence the entire incision and not just the hernia must be covered with mesh
Level 5	Some surgeons consider that suture fixation of mesh is mandatory in laparoscopic ventral hernia repair to avoid a higher recurrence rate
Level 5	Some surgeons believe that total intraperitoneal fixation with tacks reduces the surgical time, avoids parietal vascular injuries and postoperative pain, and maintains a similar recurrence rate

##### Recommendations

**Table:** 

Grade B	A mesh repair should be used in all eligible patients with a hernia defect larger than 2 cm
Grade B	For suprapubic hernias, the whole preperitoneal space should be dissected; a mesh overlap of at least 5 cm should be achieved; and fixation of the lower margin of the mesh under direct vision to Cooper’s ligaments should be performed
Grade B	Sufficient overlap of the mesh from the hernia margin and dual methods of fixation should be used

##### Discussion

Some patients are more susceptible to recurrence due to inherently weak native tissue and a proven defect of collagen synthesis [129, 130]. The recurrence rate increases with the size of the primary hernia defect: the larger the size (>10 cm), the higher is the risk of recurrence. Patients with underlying disorders such as obesity, chronic COPD, chronic cough, or diabetes mellitus are more prone to recurrence [6, 87]. Smokers with earlier failed repair attempts [131] or patients with a history of previous failed repair also contribute to the recurrence rate [132].

Conventional hernia repair by suture approximation has a high recurrence rate of 54–63 %, which decreases to 32 % with the use of mesh [133, 134]. Insufficient coverage of the incision scar also is a risk factor for recurrence after laparoscopic repair of ventral and incisional hernia [135]. Dual mesh is reported to increase the risk of recurrence [136].

Postoperative factors contributing to the recurrence after ventral or incisional hernia repair include SSI, mesh infection, wound infection, deep abscesses, and gastrointestinal complications [137].

Most common causes for the recurrence include mesh overlap less than 3 cm, displacement of the mesh, mesh contraction, and invagination into the hernia defect [82]. Improperly placed transfascial sutures together with large suture bites of mesh cause excessive tension and ultimately a hole in the mesh, which results in recurrence [138]. Mesh shift also may be a precursor to hernia recurrence, beginning with intraoperative mesh shift, followed by additional accentuating factors such as mesh contraction [139]. Most surgeons report using both transfascial sutures and laparoscopically placed tacks to secure prostheses in laparoscopic ventral hernia repair, but no firm evidence shows that this reduces the hernia recurrence rate significantly [120].

Increased intraabdominal pressure also predisposes to recurrence. This accounts for the increased recurrence in patients with morbid obesity, COPD, or chronic cough [6, 87].

Recurrence after repair can be minimized by taking precautions for patients at high risk for recurrence. Patients with conditions such as COPD and chronic cough should be treated preoperatively, and for morbidly obese patients, larger mesh should be used. Because mesh repair decreases the incidence of recurrence by half, it should be performed for all eligible patients with a hernia defect larger than 2 cm [133, 134, 140]. Laparoscopic approaches should be considered in preference to open repair because these approaches decrease the recurrence rate.

Recurrences also can be prevented by using increased overlap of the biomaterial and by placing dual devices of fixation [141]. For suprapubic hernias, mesh overlap of at least 5 cm and fixation of the mesh’s lower margin under direct vision to Cooper’s ligaments confers increased strength and durability to the repair and thus contributes to low hernia recurrence rates [142]. In addition the whole incision and not just the hernia must be repaired to reduce risk of recurrence [135]. In conclusion, applying proper technique and addressing the patients’ underlying risk factors can significantly reduce hernia recurrence.

## Section 6: comparison of open and laparoscopic repairs: operating room time, bowel injury, seroma, and wound infection

### M. Rohr, J. Lang

*Search terms.* “open” AND “laparoscopic” AND “incisional” AND “hernia”; “open” AND “laparoscopic” AND “ventral” AND “hernia”

A systemic search of the available literature was performed in August 2011 based on Medline, PubMed, and the Cochrane Library, as well as relevant journals and reference lists using the aforementioned search terms. The first search culled 322 relevant articles. In a second-level search, 339 articles were added. Altogether, 501 articles were identified, but only 59 articles were relevant, and 38 formed the basis for this systematic review.

Key questions

- Is there a difference regarding operating time?
- Is there a difference regarding frequency of bowel injury?
- Is there a difference regarding frequency of seroma formation?
- Is there a difference regarding frequency of wound infection?

#### Statements

##### Operating room time

**Table:** 

Level 1A	The open and laparoscopic techniques do not differ
Level 1B	Some studies show longer and others shorter operating room (OR) time for the laparoscopic technique. The results are inconclusive

##### Bowel injury

**Table:** 

Level 1A	The laparoscopic approach carries a higher risk for bowel injury

##### Seroma

**Table:** 

Level 1 A	The results are heterogeneous, showing no significant difference between the open and laparoscopic techniques

##### Wound infection

**Table:** 

Level 1 A	The laparoscopic approach has a significantly lower risk for wound infections

##### Recommendations

**Table:** 

Grade A	Laparoscopic repair is preferred because of a significantly reduced risk of surgical-site infection

No recommendations for OR time or seroma are possible from the current reported data.

##### Introduction

Since its introduction, laparoscopic hernia repair for ventral and incisional hernia has gained increasing popularity. Many RCTs comparing open and laparoscopic procedures have been published, enabling comparison of the two approaches. This review concerned questions relating to differences in OR time, incidence of bowel injury, seroma, and wound infection.

##### Discussion

The reported results concerning OR time are variable and inconsistent, with no statistically significant difference between the two surgical approaches. Altogether the systematic review identified six level 1a studies [2, 16, 92, 146, 147, 152], nine level 1b studies [3, 19, 109, 112, 116, 121, 143–145], one level 2a study [64], five level 2b studies [87, 113, 148–150], one level 2c study [151], six level 3 studies [108, 153–157] and one level 4 [5] study comparing OR time between the two approaches, with variable and contradictory results (Table 2).

**Table 1:** Level 1a studies comparing laparoscopic and open repairs of incisional ventral hernia

Author	No of patients lap/open	Follow- up (months	Hospital stay (days) lap/open	Return to activity/work (days) lap/open	Cost lap/open)	QOL lap/open)	Acute pain lap/open	Chronic pain lap/open	Recurrence lap/open
Sains et al. [152] 5 RCTs	366 183/183	2–27	Shorter hospital stay in lap SMD 1.82 95 % CI 3.21 to −0.44 *p* = 0.01	NR	NR	NR	No difference 95 % CI −0.41 to 0.33 *p* = 0.84 (2 trials)	NR	RR: 1.0 95 % CI 0.31−3.2 *p* = 1
Forbes et al. [16] 8 RCTs	517 269/253	23	1.1–5.7/1.33–9.06 6 RCTs Shorter hospital stay in lap	13/25 *p* = < 0.005 (1 study)	NR	NR	NR	NR	3.4 %/3.6 % RR 1.02 95 % CI 0.41−2.54 *p* = 0.56
Sauerland et al. [2] 9 RCTs	880 446/434	2–27	1.1–5.7/1.33–9.06 *p* = 0.56 6 RCTs Shorter hospital stay in lap	No difference 9% CI −2.1 to 0.7 *p* = 0.33 (2 studies)	95 % CI 1.84–3.14 *p* < 0.00001 (1 study)	No difference *p* = 0.11 (1 study)	95 % CI:P −0.45 to 0.62 *p* = 0.75 (4 studies)	95 % CI −0.24 to 1.11 *p* = 0.2 (1 study)	RR: 1.22 95 % CI 0.62–2.38 *p* = 0.56

*Lap* laparoscopic group, *QOL* quality of life, *RCT* randomized controlled trial, SMD, *CI* confidence interval, RR, *NR* not recorded

Because OR time is easy to measure, the problems seem to lie in the standards and quality of the reported studies and the different techniques (suture, stapler) used in the evolution of the laparoscopic technique between 1999 and 2011. Other possible factors accounting for these variable results include different rates and extents of adhesiolysis and varying patient-related factors.

Similar unclear data were obtained with regard to bowel injury. Although Sauerland et al. [2] noted a possible increase in bowel injury in the laparoscopic group, this was not significant.

We found three level 1a studies [2, 16, 152], four level 1b studies [3, 19, 109, 116], four level 2b studies [71, 113, 148, 149], one level 2c study [158], and two level 3 studies [108, 155] comparing the bowel injury rates between open and laparoscopic hernia repairs. The level 2c study grouped bowel injury together with visceral obstruction [158], and one level 1a study reported only overall complications [92]. Only one study reported more bowel injuries in the laparoscopic group (OR 2, 19), but the significance of the difference was low (*p* = 0.88) [152]. The remaining studies reported too few injuries (0–5), but all were small retrospective series. Nine studies reported more bowel injuries in the laparoscopic group [2, 3, 16, 19, 73, 116, 149, 152, 155], and three studies reported the same rates [108, 113, 148]. One study reported no injuries at all [109], and none reported more bowel injuries for open surgery. Thus it appears that laparoscopic hernia repair poses a greater risk of bowel injury, but clearly, more data are needed, and the increased compared with the open approach is low and acceptable.

A different picture emerged with regard to wound infections. Of the 29 studies (four level 1a studies [2, 16, 64, 153], nine level 1b studies [3, 62, 109, 112, 116 ,143, 160], seven level 2b studies [73, 87, 113, 148–150, 160], one level 2c study [151], seven level 3 studies [108, 154–156, 161–163], and one level 2c study [158]), one study reported no infection [116]. Another study reported the infection rate for both approaches [150], and the remaining studies all reported a reduced wound infection rate in the laparoscopic group (14 showing significance, including all the level 1a studies). Therefore, we can conclude that laparoscopic hernia repair is attended by a lower wound infection rate than open hernia repair, which clearly is important because it may lead to mesh infection with disastrous consequences (Table 3).

**Table 2:** Level 1b studies comparing laparoscopic and open repairs of incisional and ventral hernia

Author for all citations here	No. of patients lap/open	Mean follow up (months)	Hospital stay (days) lap/open	Return to activity/work days (range) lap/open	Cost lap/open	QOL lap/open	Acute pain lap/open	Chronic pain lap/open	Recurrence lap/open
Carbajo et al. [145]	60 30/30	24	2.23/9.06 *p* ≤ 0.05	NR	NR	NR	NR	NR	0/2 *p* = 0.05
Moreno-Egea et al. [144]	22 11/11	24	1.1/5.2 *p* ≤ 0.001	NR	NR	NR	NR	NR	0/0
Barbaros et al. [19]	46 23/23	24	2.5/6.3 *p* ≤ 0.05	NR	NR	NR	1.53/1.61 *p* ≥ 0.05	NR	0/1 *p* ≤ 0.05
Misra et al. [112]	62 32/30	12.17	1.47/3.43 *p* = 0.007	NR	Rs:1,3786.90 ($288)/Rs: 1,536.66 ($32) *p* = 0.01	Patient satisfaction score: 8.27/7.6 *p* = 0.26	Day 1: 5.95/6.05 day 3: 2.33/2.16 *p* = 0.857	3 months: 6/3 (pts) 12 months 1/1	2/1 (7.4 %/4 %) *p* = 0.95
Navarra et al. [121]	24 12/12	<12	5.7/10 *p* = 0.006	NR	NR	NR	NR	NR	0/0
Olmi et al. [143]	170 85/85	24	2.7/9.9 *p* ≤ 0.005	13 (6–15)/25 (16–30) *p* ≤ 0.005	€2,700/€3,100 (theoretical calculation)	NR	NR	NR	2/1 (2.3 %/1.1 %) *p* = NS
Pring et al. [109]	54 30/24	27.5	1.46/1.33 *p* = 0.43	3.9 ± 1.3/4.6 ± 3.3 *p* = 0.33	NR	NR	6.067/6.292 *p* ≥ 0.05	NR	1/1 *p* = NS
Asencio et al. [116]	84 45/39	12	3.46/3.33 *p* = 0.78	NR	NR	RR −0.04, CI −0.10 to 0.01, *p* = 0.11	Day1: 47.66/42.86 *p* = 0.285 Day7: 28.93/22.55 *p* = 0.205	3 Months: 13.51/7.10 *p* = 0.056 12 months 10.31/6.00 *p* = 0.201	9.7 %/7.9 % *p* = 0.77
Itani et al. [3]	146 73/73	24	4.0/3.9 *p* = 0.91	23.0/28.5 *p* = 0.06)	NR	No difference in improvement at 8 wks *p* = 0.17 for pain at rest *p* = 0.07 for worst pain	NR	NR	9/6 (12.5 %/8.2 %) *p* = 0.44

*Lap* laparoscopic, *QOL* quality of life, *NR* not recorded, *Rs* randomized study, *NS* nonsignificant difference

The reported finds on the incidence of seroma again are heterogeneous, as shown in the following chart:

Study Level More seroma Less seroma Same rate

1A 3 [116, 152] 1 [16]

1B 4 [112, 143] (2s [19, 116]) 3 [3, 109, 145](1 s [62])

2A 1s [64]

2B 2 [113] (1s [73]) 3 [87, 148, 149] 1 [151]

3 5 [108, 154–156, 162] 1 [161]

s = significant (stated for laparoscopic hernia repair)

Unfortunately, in the published literature, seroma is not reported uniformly (i.e. some articles report only symptomatic seroma). A commonly accepted definition of seroma types is needed. The new classification [164] should address this problem and thus result in more meaningful reports.

##### Comment

Overall, the published experimental and clinical studies outline an unclear picture compounded by differences in techniques used (stapler, sutures) and levels of experience. These account for the variability of reported data such as OR times. In essence, high-quality, high-volume studies are too few. The reported studies often do not differentiate between ventral, umbilical, and incisional hernias, rendering interpretation of the outcomes more difficult. In addition, complications often are grouped together, making it difficult to analyze specific complications.

##### Conclusions

The main important finding is that laparoscopic hernia repair results in a lower incidence of wound infection than open repair. For other complications such as bowel injury, long OR time, and seroma, no clear statement is possible because of conflicting data and poor levels of evidence.

### Comparison of hospital stay, return to activity, cost, quality of life, pain, and recurrence after laparoscopic and open ventral and incisional hernia repair

#### M. C. Misra, V. K. Bansal, P. P. Prakash, D. Babu, P. Singhal, R. H. Fortelny

*Search terms:* “hospital stay” AND “laparoscopic incisional hernia repair” AND “ventral hernia” AND “LIVHR” AND “length of stay” AND “laparoscopic vs open incisional hernia” AND “defect size” AND “primary ventral hernia” AND “fixation” AND “sutures” AND “tackers” AND “recurrent incisional hernia”

The databases used for the search included Pubmed, the Cochrane database, Medline. The search also included relevant journals and reference lists in the English language until September 2011. The search yielded 122 publications, 25 of which were relevant to the search questions. These were supplemented by 40 articles found by manual searches, making a total of 65.

##### Statements

**Table:** 

Level 1a	Laparoscopic incisional and ventral hernia repair (LIVHR) significantly reduces hospital stay compared with open repair
Level 1b	Hospital stays are comparable after suture fixation and tacks fixation
Level 2b	The hospital stay is significantly shorter after LIVHR than after open repair for patients with hernias larger than 15 cm
Level 3	The hospital stay is shorter after LIVHR for primary ventral hernia than after incisional hernia

##### Recommendations

**Table:** 

Grade A	Based on the shorter hospital stay, LIVHR is the preferred operative technique

### Hospital stay

Laparoscopic incisional hernia repair is associated with a shorter hospital stay than open repair. Three level 1a studies compared the hospital stay between open and laparoscopic incisional and ventral hernia repairs. In the 2011 Cochrane review [2], six [19, 112, 121, 143–145] of the nine trials [3, 19, 109, 112, 116, 121, 143–145] reported a significantly shorter hospital stay after laparoscopic repair than after open repair (5.7 vs. 10 days). Forbes et al. [16] (eight RCTs) and Sajid et al. [92] (five RCTs) reported similar findings in their metaanalyses. One level 1b study compared the hospital stay between suture and tacks fixation and found the results to be comparable (1.13 vs. 1.16 days; *p* = 0.77) [165].

Four level 1a/2a studies [2, 6, 7, 52] analyzed hospital stay. The mean hospital stay was shorter in six RCTs [19, 112, 121, 143–145] and five nonrandomized studies [52, 73, 113, 157, 167]. The overall hospital stay was shorter in laparoscopic incisional and ventral hernia repair (2 vs. 4 days; *p* = 0.02) [147] (Table 4).

**Table 3:** Level 1a/2a studies comparing laparoscopic and open repairs of incisional and ventral hernia

Author for all citations here	No. of patients lap/open	Follow up (months) mean/range	Hospital stay (days) lap/open	Return to activity/work (days) Lap/open	Cost lap/open	QOL lap/open	Acute pain (%) lap/open	Chronic pain (%) lap/open	Recurrence lap/open
Cassar et al. [205] (19 studies)	1,896 1,598/298	6–53	NR	NR	NR	NR	NR	1.8 (4 studies)	0–9 %/0–10 %
Goodney et al. [147]	712 322/ 390	NR	2/4 *p* = 0.02	NR	NR	NR	NR	NR	NR
Rudmik et al. [210] (10 studies)	2,060 all lap	34	3.2/2.2 (tacks vs. suture)	NR	NR	NR	NR	NR	4.5/4.4 (tacks vs suture)
Sains et al. [152] (4 studies)	351,148/203	1–85	RR 3.2 95 % CI −5.4 to 1.15 *p* = 0.003	NR	NR	NR	NR	NR	0.06
Pierce et al. [52] (45 studies)	5,340 4,582/758	17–25	2.4/4.3 *p* = 0.0004	NR	NR	NR	NR	1.0/0.9 *p* = 0.93	4.3 %/12.1 % *p* ≤ 0.0001
LeBlanc et al. [209] (12 studies)	3,434 All lap with or without sutures	6–49	NR	NR	NR	NR	NR	NR	1.8 %/4 % (with and without sutures)
Bedi et al. [207] (34 studies)	3,266 all lap	29.7	NR	NR	NR	NR	2.75	NR	3.67 %
Müller-Riemenschneider et al. [17] (15 studies)	2008 906/1,102	1–24	3.4	NR	NR	NR	NR	3.6/4.1 *p* > 0.05	0–20.7 %/0–35 %
Pham et al. [146] (9 studies)	1,066 497/569	2–24	0.8–3.4/1.5–9.06	NR	NR	NR	NR	6.1/4.1	0–13 % 0–20.7 %
Total	20,073 1,6753/3,320	1–85	2.4/4.3	NR	NR	NR	NR	3.6/3	0–20.7 %/0–35 %

*Lap* laparoscopic, *QOL* quality of life, *NR* not recorded

**Table 4:** Level 2b/3 studies comparing laparoscopic vs open repair of incisional and ventral hernia

Author for all citations here	No of patients lap/open	Mean follow-up (months) lap/open	Hospital stay days (range) lap/open	Return to activity/work (days) lap/open	Cost ($) lap/open	QOL lap/open	Acute pain lap/open	Chronic pain lap/open	Recur-rence lap/open (%)
Park et al. [157]	105 49/56	24.1/53.7	3.4/6.5 *p* < 0.001	NR	NR	NR	NR	NR	21/3
DeMaria et al. [167]	39 21/18	12–24	0.8/4.4 *p* < 0.05	NR	Initial and readmission cost 8,273 ± 2,950/12,461 ± 5,987 *p* < 0.05	NR	Analgesic use 10 versus 79 % *p* < 0.05	NR	5/0
McKinlay et al. [208]	170 69 Recurrent 101 Primary	19/27	NR	NR	NR	NR	NR	2.8 %/0 % in recurrent versus primary	7 Recur-rent 5 Primary *p* = 0.53
Lomanto et al. [113]	100 50/50	20.8	2.7/4.7 *p* = 0.044	NR	NR	NR	VAS 2.9/4.1 *p* = 0.001	NR	2/10
Olmi et al. [154]	50 25/25	9/24.5	NR	NR	NR	NR	NR	NR	2/0
Bingener et al. [73]	360 127/233	36/25	0.9 ± 1.4/1.4 ± 2.0 *p* = 0.01	NR	NR	NR	NR	NR	9/12 *p* = 0.36
Ching et al. [211]	168 all lap 42 mor-bidly obese/124 nonobese	19	NR	NR	NR	NR	NR	Obese/nonobese 5 %/10 % *p* = 0.5	Obese/nonobese 10/13 *p* = 0.78
Ceccarelli et al. [212]	181 94/87	38	NR	NR	NR	NR	NR	NR	2.1/6.9 *p* > 0.05
Kurmann et al. [87]	125 69/56	32.5/65	6 (1–23)/7 (1–67) *p* = 0.001	21/42 *p* > 0.05	NR	NR	NR	VAS 0.5/0.6 *p* > 0.05	18/16 *p* = 0.6
Total	960 435/525	24.8/34.4 (7 studies)	2.7/4.6 (5 studies)	21/42 (1 study)	8,273 ± 2,950/12,461 ± 5,987 (1 study)	NR	2.9/4.1*p* = 0.001 (1 study)	0.5/0.6 *p* > 0.05 (1 study)	8.4/6.8 (7 studies)

*Lap* laparoscopic, *QOL* quality of life, *NR* not recorded, *VAS* visual analog scale

Ten level 3 studies [5, 87, 148, 150, 154, 155, 163, 168–170] reported a hospital length of stay and a follow-up evaluation ranging from 4 to 44 months. The hospital stay ranged from 3 to 8.1 days for open repair and 2.1 to 6 days for laparoscopic repair. Five studies showed a significantly shorter hospital stay in the laparoscopic repair group [150, 154, 155, 163, 169].

In 34 level 4 noncomparative studies [11, 20, 24, 45, 79, 82, 102, 171–197] the reported postoperative hospital stay ranged from 1 to 17 days after laparoscopic repair. Kua et al. [179] reported that 57 % of their patients were discharged the next day after surgery, with another 27 % discharged within 48 h. Raftopoulos et al. [180] reported a significantly shorter hospital stay after laparoscopic repair of primary ventral hernia than after incisional hernia repair (0.6 vs. 2.2 days; *p* = 0.03).

#### Comments

The hospital stay is shorter after laparoscopic incisional and ventral hernia repair than after open repair. Few studies have compared the hospital stay after laparoscopic incisional and ventral hernia repair in relation to defect size, and no available study has reported data for hospital stay with respect to the type of mesh fixation and type of mesh.

### Return to activity

*Search terms*: “return to work” AND “laparoscopic incisional hernia” AND “ventral hernia repair” AND “return to activity” AND “mesh fixation” AND “suture” AND “tacker” AND “defect size” AND “defect site” AND “recurrent incisional hernia” AND “type of mesh”

The search yielded eight publications, only three of which were relevant to the search questions, and a further five publications were found by manual search.

#### Statements

**Table:** 

Level 1a	The time until return to activity does not differ significantly between laparoscopic and open repairs
Level 1b	Laparoscopic incisional hernia repair is associated with a faster return to work than open repair
	Suture fixation is associated with a faster return to work after laparaoscopic repair than after tacks fixation
Level 2b	Return to activity after laparoscopic incisional and ventral hernia repair does not differ significantly between suture and tacks fixations
Level 4	The time until smokers and patients with hard physical work demands can return to work is significantly longer

##### Recommendations

**Table:** 

Grade A	Suture fixation is recommended over tacks plus suture fixation because of early return to full activity
Grade B	Because of the earlier return to work, LIVHR is preferred to open repair

Return to daily activities or work is an important measure for assessment of any surgical intervention. The Cochrane review [2] included two RCTs with reports on return to activity. Pring et al. [109] reported no significant difference in return to activity, whereas Itani et al. [3] reported that the time to resumption of work was shorter for the laparoscopic group (median, 23.0 vs. 28.5 days; *p* = 0.06).

From an RCT, Olmi et al. [143] reported that patients in the laparoscopic group could return to work in a significantly shorter time (13 vs. 25 days; *p* = 0.005). From a level 2b study, Kurmann et al. [87] reported an earlier return to work after LIVHR, but the difference between the two groups was not significant (21 vs. 42 days; *p* > 0.05).

From a level 3 study Raftopoulos et al. [150], reported significantly earlier return to work after LIVHR (25.9 vs. 47.8 days; *p* = 0.036), although the mean time to resumption of activity did not differ significantly. Six level 4 studies [171, 177, 178, 195, 198, 199] reported return to activities after LIVHR. Kua et al. [179] reported that 82 % of patients returned to household duties within 1 week after laparoscopic repair. Eriksen et al. [101] concluded that the time until smokers and patients with hard physical work demands could return to work was significantly longer.

##### Return to activities after suture versus tacks fixation

In their RCT, Bansal et al. [165] reported a significantly shorter time until resumption of activity after suture fixation than after tacks fixation (*p* < 0.001). In their level 2b study, Nguyen et al. [96] reported no significant difference in time until return to activity after the two fixation techniques (respectively 50 vs. 42 % of the patients after 1 week).

##### Comments

Few RCTs compare return to work. Time until return to work is the same or shorter after laparoscopic compared with open repair. No available study reports on return to activity considering different methods of mesh fixation, mesh types, and defect characteristics. More RCTs are needed to analyze different aspects of laparoscopic repair such as fixation method, mesh type, and defect characteristics.

### Cost

#### *Search terms:* “cost,” “laparoscopic incisional hernia repair,” “laparoscopic ventral hernia repair”

##### Statements

**Table:** 

Level 1a	The cost of surgery is higher for laparoscopic procedure, but a shorter hospital stay may make laparoscopic surgery more cost effective
Level 1b	Suture fixation is a cost-effective alternative to tacks fixation for small and medium-sized defects in anatomically accessible areas
	Open repair is nine times cheaper than laparoscopic repair
	A shorter hospital stay is likely to reduce the total direct hospital cost
Level 3	Laparoscopic repair is costlier than open repair in terms of hospital cost but has a decreased mean overall cost
Level 5	A self-adhering prosthesis may decrease the cost of these procedures

##### Recommendations

**Table:** 

Grade A	Suture fixation in laparoscopic incisional hernia repair is recommended
Grade D	Laparoscopic incisional hernia repair can be recommended as a cost-effective repair

The reports in the literature regarding comparison of costs between laparoscopic and open repairs are conflicting. The laparoscopic approach has been shown to result in higher operative costs but better cost effectiveness because it is associated with significantly lower mortality, reduced morbidity, fewer intensive care unit (ICU) admissions and 30-day readmissions, shorter hospital stay, and thus significantly reduced hospital costs.

The search identified 42 studies analyzing costs in incisional and ventral hernia repair, but only 14 papers were considered relevant. In the Cochrane review [2], only one study by Misra et al. [112] performed an economic analysis comparing open and laparoscopic repairs. That study found open repair to be nine times cheaper than laparoscopic repair. Theoretical calculation by Olmi et al. [143] showed the cost of laparoscopic surgery to be higher than the cost of open repair (1,900 vs. 300 euros) but the overall cost to be less than that of the open technique, probably due to a shorter hospital stay.

Three prospective studies [96, 167, 198] comparing cost showed laparoscopic repair cheaper. DeMaria et al. [167] attributed the lower costs to lower readmission rates, whereas Earle et al. [153], attributed the lower costs to a shorter hospital stay and a lower readmission cost.

Four retrospective comparative studies comparing laparoscopic and open repairs [87, 150, 154, 198] in terms of cost showed that the direct costs of hernia repair surgery in terms of longer OR time and cost of equipment and materials, is higher in laparoscopy group but that the overall costs are lower or equivalent due to a shorter hospital stay and lower complication rates.

Bencini et al. [155] showed that despite a higher mesh cost, laparoscopic repair was cheaper due to the shorter hospital stay. Beldi et al. [148] also showed similar results. These findings were complemented in two other studies by Holzman et al. [168] and Wright et al. [200].

Many variables such as mesh type, fixation technique, and technique of repair come into play in cost calculation. Any modification of these cost variables influences the overall cost of either procedure. An RCT published by Bansal et al. [106, 165] comparing suture mesh fixation with tacks mesh fixation showed that the overall cost for suture fixation was significantly less than for tacks fixation of small to medium-size defects ($575.42 more expensive; *p* = < 0.001).

Type of mesh also dictates the overall cost. A prospective study by Alkhoury et al. [201] comparing costs and clinical outcomes with the use of non-heavyweight PP mesh and other meshes showed that PP meshes were substantially cheaper. The cost saving was $436 per patient with Proceed (Ethicon Inc. Somerville, NJ, USA), $770 per patient with Composix (Davol, Warwick, RI, USA), and $931 per patient with ePTFE mesh. In a retrospective comparative study by Bencini et al. [155], PP mesh was significantly cheaper than ePTFE mesh.

##### Comments

None of the reported studies showed full economic evaluation focused on the relevant alternatives. The studies did not primarily aim to investigate costs or cost effectiveness. The cost analysis studies reported to date were inadequate, so proper health technology assessment (HTA) studies are needed to address cost efficacy and cost utility.

### Quality of life

*Search terms:* “quality of life” AND “laparoscopic” AND “incisional hernia” AND “ventral hernia repair” AND “open incisional hernia repair” AND “patient satisfaction” AND “cosmesis” AND “mesh fixation” and “suture” AND “tacker” AND “defect size” AND “recurrent incisional hernia”

The search resulted in 27 publications, but only seven were relevant to the search question. An additional four publications were identified by manual search, giving a total of 11 publications for the study.

#### Statements

**Table:** 

Level 1a	Quality of life (QOL) does not differ between open and laparoscopic repairs of incisional and ventral hernia
Level 1b	Use of absorbable sutures with tacks leads to better QOL than tacks with nonabsorbable sutures or tacks only
	The QOL does not differ between suture and tacks fixation in laparoscopic repair of incisional and ventral hernia
Level 2b	Laparoscopic repair leads to significant improvement in QOL compared with open repair
Level 4	Laparoscopic ventral hernia repair leads to a significant improvement in QOL experienced by the patient
	Patient satisfaction is higher after laparoscopic ventral hernia repair than after open repair
Level 5	Patients are satisfied cosmetically after suture fixation

##### Recommendations

**Table:** 

Grade A	Laparoscopic repair is recommended because it gives a QOL comparable with that of open repair

In recent years, the QOL experienced by patients has become an essential evaluation parameter for chronic illness and chronic morbidity and is increasingly used in decisions related to treatment strategies.

##### QOL in open versus laparoscopic ventral hernia repair

In the Cochrane review [2], only two of nine RCTs compared QOL, with these two RCTs reporting no significant difference in QOL between open and laparoscopic ventral hernia repairs [3, 116]. Patient satisfaction with cosmetic appearance was studied in only one of the trials [112], and no significant difference in cosmetic outcomes was reported (*p* = 0.26).

In a level 2b study, Mussack et al. [202] found no significant difference in any domains of the Medical Outcomes Questionnaire-Short Form (SF-36) questionnaire. In contrast, Hope et al. [100] reported improved postoperative QOL in four of the eight SF-36 domains (general health, vitality, role emotional, mental health) with laparoscopic repair versus open repair. These authors also measured QOL with the Carolinas Comfort Scale and reported improvement in all physical variables with laparoscopic repair versus open repair.

Uranues et al. [72] and Eriksen et al. [101] (level 4 study) reported substantially improved health-related QOL after laparoscopic incisional hernia repair. Whereas Uranues et al. [72] reported significant improvement in three of the five Gastrointestinal Quality of Life Index (GIQIL) domains (symptoms, emotional function, and physical function), Eriksen et al. [101] measured the SF-36 domains and found improvement in general well-being, body pain, and fatigue.

##### QOL and fixation technique

Different fixation techniques (suture or tacks) may be associated with varying degrees of early postoperative and chronic pain and may affect QOL postoperatively. Bansal et al. [165] in an RCT compared QOL after fixation with either sutures only or tacks and found no significant difference between the two groups. Wassenaar et al. (RCT) [203] evaluated QOL after three fixation techniques: tacks plus absorbable sutures, double crown of tacks, and tacks plus nonabsorbable sutures. They found that the tacks plus absorbable sutures group was significantly better than the double-crown tacks group in physical functioning and role limitation due to emotional problems (*p* = 0.02).

##### Patient satisfaction in laparoscopic ventral hernia repair

Patient satisfaction is an indicator of postoperative QOL and cosmetic outcomes. Only two studies commented on patient satisfaction. Bansal et al. [106, 165] from an RCT of suture versus tacks fixation in laparoscopic repair reported that patients were satisfied cosmetically after suture fixation but that the patient satisfaction scores did not differ significantly between suture and tacks fixation. Perrone et al. [24] reported that the patient satisfaction score was high after laparoscopic incisional hernia repair.

##### Comments

Few RCTs have compared QOL between laparoscopic and open ventral hernia repair. Very few studies have compared QOL for different aspects of laparoscopic ventral hernia repair such as type of mesh, fixation method, and defect characteristics. Different methods have been used for QOL assessment in different studies, making analyses and comparisons difficult. More RCTs are needed to evaluate different parameters of laparoscopic ventral hernia repair using one standardized method.

### Pain

*Search terms*: “pain” AND “laparoscopic” AND “incisional hernia repair” AND “ventral hernia” AND “LIHVR” AND “mesh fixation” AND “suture” AND “tackers” AND “type of mesh” AND “factors for pain” AND “defect size” AND “defect site” AND “pain” AND “acute pain” AND “chronic pain” AND “recurrent incisional hernia” AND “preoperative pain” AND “postoperative pain”

The search yielded 113 publications, 39 of which were relevant to the search question, and a manual search yielded another ten papers, resulting in a total of 49 publications used for the review.

#### Statements

**Table:** 

Level 1a	The incidence of pain, both acute and chronic, does not differ significantly different open and laparoscopic ventral hernia repairs
Level 1b	In laparoscopic repair, the incidence of early postoperative pain and chronic pain is less with suture fixation than with tacks fixation
	Chronic pain in laparoscopic ventral hernia repair is not significantly associated with preoperative pain
	Pain does not differ between heavyweight PP mesh and lightweight barrier-coated meshes
Level 2b	Chronic postoperative pain is more common after laparoscopic ventral hernia repair in recurrent cases than in primary cases
Level 4	Fixation with both tacks and transfixation suture results in more pain
	Pain after laparoscopic ventral hernia repair is mostly at the suture site
	Defect closure may lead to chronic pain
Level 5	Sutures cause ischemic injuries to the anterior abdominal wall musculature or the neurovascular bundle, resulting in pain. Nerve entrapment by tacks is another possible explanation for the postoperative pain

##### Recommendations

**Table:** 

Grade A	The pain scores associated with laparoscopic and open ventral hernia repairs are similar
Grade A	Suture fixation alone for small and medium-sized defects may result in less pain and can be recommended

Postoperative pain rather than recurrence is the most important outcome measure after laparoscopic incisional and ventral hernia repair. The use of transfascial sutures and tacks can cause substantial early postoperative pain and chronic pain even months or years after surgery. Consequently, current interest focuses increasingly on the genesis of pain after laparoscopic ventral hernia repair and methods to reduce such pain. Various factors responsible for chronic pain have been cited including type of mesh fixation, defect closure, recurrent incisional hernias, and type of mesh.

##### Acute pain

Two systematic reviews of RCTs (level 1a) report on postoperative pain after laparoscopic versus open incisional hernia repair. The Cochrane review [2] (metaanalysis of ten RCTs), comprising 880 patients, included four RCTs (Asencio et al. [116], Barbaros et al. [19], Misra et al. [112] and Pring et al. [109]) that measured pain after surgery, and in all RCTs, the intensity of pain was similar between the open and laparoscopic repair groups. Sajid et al. [92] analyzed five RCTs, and reported similar findings of no difference in overall postoperative pain between laparoscopic and open repairs (*p* = 0.84).

##### Chronic pain

The incidence of chronic pain after laparoscopic incisional and ventral hernia repair is reported to range from 1 to 3 % [204]. Only two RCTs reported on chronic pain in laparoscopic ventral hernia repair versus open repair. Asencio et al. [116] in a level 1b study reported no significant difference in mean pain scores in follow-up assessments at 3 months and 1 year. Also in a level 1b study, Itani et al. [3] reported that the mean worst pain after 1 year was significantly less in the laparoscopic group (15.2 mm lower on a visual analog score of 0–100 mm), but the mean pain score values for both groups are not included.

Three systematic reviews of nonrandomized studies (level 2a) were identified. These reviews by Pierce et al. [52] (review of 14 paired and 31 unpaired studies), Müller-Riemenschneider et al. [17] (review of 14 comparative studies), and Cassar et al. [205] (review of 19 studies) included a total of 9,244 patients (2,102 open and 7,384 LIVHR procedures) followed up for a mean period of 24 months after open repair and 17.3 months after laparoscopic ventral hernia repair. Pierce et al. [52] and Müller-Riemenschneider et al. [17] reported no difference in chronic pain between laparoscopic and open repairs. Cassar et al. [205] reported the mean incidence of chronic pain to be 1.8 % in 4 of 19 studies.

In 15 level 4 studies [11, 20, 22, 24, 79, 102, 186, 187, 190, 193–195, 198, 201, 206], the incidence of chronic pain for 4,236 patients during a follow-up period ranging from 6 to 64 months varied from 1 to 14.7 %. Heniford et al. [187] reported that pain was mostly at the suture site. Sharma et al. [82] reported that more pain occurred after the use of both tacks and transfixation sutures.

##### Pain and type of fixation: suture or tacks?

The pain in laparoscopic incisional and ventral hernia repair is related to mesh fixation with either tacks or sutures. The pain due to fixation differs from that at port sites.

Three RCTs (level 1b) studied the association of pain with the type of fixation. Wassenaar et al. [203] conducted an RCT of three fixation techniques (tacks with absorbable sutures, nonabsorbable sutures, and only tacks) and found no significant difference in VAS scores among the three techniques of mesh fixation at any time during a follow-up period of 3 months. On the other hand, Bansal et al. [106] reported higher pain scores in the tacks fixation group than in the suture fixation group during the early postoperative period, which became insignificant at 3 months and during further long-term follow-up assessment [167]. In an RCT during 211, Beldi et al. [105] also did not find any significant difference in VAS scores between tacks and suture fixations during 6 months of follow-up evaluation.

Three nonrandomized comparative studies (level 2b) reported chronic pain after suture and tacks fixation. Nguyen et al. [96] reported no significant difference between the two fixation groups in their nonrandomized comparative trial comparing suture and tacks. Beldi et al. [148] and Kurmann et al. [87] reported that pain after laparoscopic ventral hernia repair is mostly at the transfixation suture site.

In four noncomparative trials (level 4) [6, 82, 84, 105] consisting of 2,649 patients and follow-up periods ranging from 1 to 120 months, the incidence of chronic pain was 16.4 % in the suture groups and 12.7 % in the tacks groups. Chronic pain was highest for patients in whom both tacks and sutures were used (Sharma et al. [82]). However, in a study by Chelala et al. [102] using transfascial suture fixation only, 97.5 % of the patients were pain free. Seven of the patients (1.75 %) reported chronic pain, which resolved gradually, and only three patients (0.75 %) required excision of a neuroma at the suture fixation site.

Bedi et al. [207] in a review of 34 original studies commented that sutures for mesh fixation might cause ischemic injuries to anterior abdominal wall musculature or neurovascular bundle, resulting in pain. Nerve entrapment due to a tack is another possible explanation for postoperative pain (Level 5).

##### Association of chronic pain

In a level 2b study, McKinlay et al. [208] analyzed the incidence of chronic pain after laparoscopic ventral hernia repair of primary and recurrent incisional hernias and reported chronic postoperative pain during more than 6 months. Their report of 101 primary cases showed two cases (2.8 %) of recurrent pain verses 69 cases of no chronic pain.

The mesh material also may play an important role in the causation of pain. In a level 1b study, Bansal et al. [106, 165] investigated the association of acute and chronic pain with the type of mesh and did not find any difference in pain scores between heavyweight PP mesh and lightweight barrier coated meshes.

The efficacy of mesh repair is based on the formation of a strong mesh aponeurotic scar tissue complex (MAST complex). But inflammation beyond the optimum range may entrap neural structures, leading to chronic pain. Currently, large numbers of lightweight composite meshes are available that are claimed to produce optimum fibrotic reaction and to decrease the incidence of chronic pain. However, not many available studies have compared the composite meshes with the PP meshes.

Chelala et al. [102] and Franklin et al. [45] reported chronic pain incidences of 2.5 and 3.1 %, respectively, after defect closure. This may indicate that closure of the defect with subsequent traction may even contribute to chronic postoperative pain.

No study was found depicting the association of chronic pain with acute pain, preoperative pain, or site and size of the defect.

##### Comments

None of the studies evaluated pain as the primary outcome. No study compared the association of pre- and postoperative pain. Few RCTs have reported pain after laparoscopic incisional and ventral hernia repair. Their comparisons mainly involve open repair. Even fewer studies have reported on chronic pain. The sample is small in all RCTs. Very few studies have evaluated the association of pain with the method of fixation or the type of mesh. No data are available regarding the relation of pain with defect site, defect size, acute pain, or recurrent hernias. More RCTs are needed with greater numbers of patients and longer follow-up periods. Larger trials also should include separate analyses of primary ventral and incisional hernias. Studies are strongly needed to assess the relation of pain with fixation, type of mesh, defect size and site, and recurrent hernias.

### Recurrence

*Search terms:* “laparoscopic incisional hernia repair” AND “LIVHR” AND “incisional hernia” AND “ventral hernia” AND “open hernia repair” AND “ recurrence rates” AND “relapse”

#### Statements

**Table:** 

Level 1a	No significant difference in recurrence is found between open and laparoscopic incisional/ventral hernia repairs

##### Recommendations

**Table:** 

Grade A	The recurrence rates for laparoscopic and open ventral hernia repair are similar
Grade B	Suture and tacks fixation are equally effective, but all suture fixation for small and medium-sized defects is more cost effective

Recurrence is one of the most important outcomes of incisional and ventral hernia repair. Recurrence depends on various patient-, hernia-, tissue-, and technique-related factors. Most of these risk factors are constant and cannot be altered, but technical factors such as type of repair, type of mesh used, method of mesh fixation, and margin of mesh overlap can be modified to potentially improve recurrence rates.

Three metaanalyses [2, 16, 92] comprising 880 patients (446 laparoscopic and 434 open repairs) compared recurrence rates for laparoscopic and open repairs. None demonstrated a significant difference in recurrence rates (relative risk (RR), 1.22; 95 % confidence interval (CI), 0.62–2.38; *p* = 0.58) after 2–68 months of follow-up evaluation. Half of these trials reported a follow-up period shorter than 2 years [2].

Forbes et al. [16] in a metaanalysis of 8 RCTS consisting of 517 patients found no significant difference in recurrence rates between laparoscopic and open repairs during a mean follow-up period of 23 months. The overall recurrence rate was low due to the small hernia size in most of the studies and the lack of a uniform definition for recurrence.

Only nine RCTs [3, 19, 109, 112, 116, 121, 143–145] have compared recurrence rates for laparoscopic and open incisional and ventral hernia repairs, and seven of these RCTs found no significant difference in the recurrence rates, whereas two studies (Carbajo et al. [145] and Barbaros et al. [19]) showed a lower recurrence rate with laparoscopic repair. Barbaros et al. [19] randomized 23 patients each to laparoscopic and open repairs and found that the recurrence rate after laparoscopic repair was significantly lower (*p* < 0.05). They had only one recurrence, which was in the open group [19]. Carbajo et al. [145], also showed a significantly lower recurrence rate in the laparoscopic group (*p* < 0.05) during a 2-year follow-up period.

Recurrences were attributed to various factors. Misra et al. [112], attributed recurrence to inadequate space for mesh fixation in a low-lying defect, whereas Olmi et al. [143], attributed recurrence to inadequate mesh overlap, and Itani et al. [3] attributed recurrence to postoperative surgical-site infection.

In eight systematic reviews [17, 52, 146, 152, 205, 207, 209, 210] of prospective studies comparing laparoscopic and open repairs for 19,421 patients, the recurrence rates ranged from 0 to 20.7 % in the laparoscopic group and from 0 to 35 % in the open group during follow-up periods of 1–85 months. Only Pierce et al. [52], showed a significantly lower recurrence rate for laparoscopic repair. These authors published a pooled data analysis of 45 studies during a period of 12 years comparing laparoscopic and open ventral hernia repairs. In these 45 studies, representing 5,340 patients (4,582 laparoscopic and 758 open repairs), laparoscopic repair was associated with a significantly lower recurrence rate (*p* < 0.0001).

Various potential causes for recurrence also have been identified. Cassar et al. [205] reviewed 19 prospective comparative studies comprising of a total of 1,896 patients (1,598 laparoscopic and 298 open repairs) and found higher recurrence rates for large hernias and patients with a wound infection. They also found that staples alone were inadequate for fixation of mesh and that the interval between two staples should be less than 1 cm. Bedi et al. [207] stated that recurrence decreases with the use of transfacial sutures and with experience.

Nine prospective comparative studies were identified [73, 113, 154, 157, 167, 208, 211, 212] with a total of 1,298 patients (773 laparoscopic and 525 open repairs). The recurrence rate ranged from 2 to 21 % after laparoscopic repair and from 0 to 16 % after open repair during a follow-up period of 9–65 months. In the two studies, the recurrence rates were significantly lower in the laparoscopic group. Bingener et al. [73] compared laparoscopic and open repairs prospectively, with 127 patients in the laparoscopic group and 233 patients in the open group, during a follow-up period of 25–36 months and reported a recurrence rate of 9 % in the laparoscopic group and 12 % in the open group (*p* = 0.36). Ceccarelli et al. [212], in a comparison of 94 patients with laparoscopic repair and 87 patients with open repair found a significantly lower recurrence rate after laparoscopic repair (*p* > 0.05) and postulated that the recurrence rate was lower because laparoscopy helps to identify defects not clinically identifiable.

Studies also have noted that lateral defects [157], larger defects [87, 213], BMI higher than 30 kg/m^2^ [208], and perioperative complications [87, 157] are associated with significantly higher recurrence rates. Patients with recurrent or multiple hernias also have shown a higher rate of recurrence, although the difference has not been statistically significant [87]. McKinlay et al. [208] compared laparoscopic repair for 69 recurrent hernias and 101 primary hernias. The recurrence rate was comparable (7 vs. 5 %), but the mean time to recurrence was shorter in the recurrent hernia group (*p* = < 0.0001).

In eight retrospective studies [5, 155, 169–171, 194, 208, 212] comprising 765 patients, the recurrence rate ranged from 0 to 15.7 % in laparoscopic group during a follow-up period of 6–40 months. Zografos et al. [171] analyzed 106 patients retrospectively (30 laparoscopic and 76 open repairs) during 40 months. The recurrence rates in the two groups were comparable (3.3 vs. 2.6 %). Ceccarelli et al. [212] postulated that the causes for recurrence in laparoscopic repair were rolling up of mesh, incomplete stretching of mesh, and incomplete covering of the defect.

A total of 56 case series [5, 6, 11, 14, 20, 21, 24, 45, 71, 72, 76, 79, 82, 84, 101, 102, 107, 155, 169–171, 173–186, 189–191, 193–199, 206, 208, 213–221] involving laparoscopic repair for 8,677 patients were identified. The recurrence rates ranged from 0 to 20 % during a follow-up period of 1–84 months.

It has been noted that recurrences commonly occur at the mesh margins along the mesh–tissue interface. This finding has been validated by an experimental study, which found that increasing the mesh overlap to 4 cm eliminated mesh disruption [186]. In many studies, a mesh overlap of 3–5 cm or more has been used, and reports have shown recurrence rates to be less than 5 % [177, 186, 197].

The study by Park et al. [157] had a recurrence rate of 11 %, but the mesh overlap was only 2.5 cm, which likely was responsible for the high recurrence rate. Theodoropoulou et al. [216] had a recurrence at the periphery of the mesh despite a 3-cm overlap. LeBlanc [209], reviewing the literature on fixation techniques, recommended that the minimum mesh overlap should be 4–5 cm if transfascial sutures are not used, and at least 3 cm when transfascial sutures are used.

Mesh size is equally important. Wassenaar et al. [193] stated that the mesh should cover not only the defect but also the entire incision to prevent recurrence. A larger mesh may protrude through the defect, causing recurrence. Mesh contraction and migration into the defect are common with a smaller mesh.

Uranues et al. [72] studied recurrence rates after laparoscopic repair of recurrent hernias and reported that the risk was similar to that for primary repair (3.5 %). Chelala et al. [102], in their series of 400 cases, noted that recurrence could be due to nonclosure of the defect with extrusion of mesh into the defect, especially when the mesh size is insufficient.

Mesh fixation is an important determinant of recurrence rates. Variable recurrence rates have been reported in the literature with the use of different mesh fixation techniques. Three RCTs comparing various fixation devices and techniques were identified. None of them showed a significant difference in terms of the recurrence rate between suture only, suture with tacks, and tacks only fixations.

Similarly, two systematic reviews with a total of 6,824 patients also were identified, which showed no significant difference between suture and tacks fixations [209, 210]. In a collective review of 23 studies and 12 comparative studies by LeBlanc [209], mesh fixation with sutures only resulted in the lowest recurrence rate (0.8 %) compared with that by tacks alone (1.5 %). Mesh fixation with tacks and sutures resulted in a recurrence rate of 3.5 % during a mean follow-up period of 22 months.

##### Studies using tacks and sutures for mesh fixation

In an RCT, Bansal et al. [165] randomized 106 patients to compare suture and tacks fixation. They reported two recurrences, both in the tacks fixation group, during a mean follow-up period of 31 months. Ben-Haim et al. [21] presented a retrospective study of 100 patients who underwent ePTFE mesh fixation with both transfascial sutures and tacks. The exact mesh fixation technique and mesh overlap size were not mentioned. The recurrence rate was 2 % during a mean follow-up period of 19 months. The proposed mechanisms of recurrence included detachment of tacks and inadequate mesh overlap.

Heniford et al. [6] published the largest series (850 patients) of laparoscopic hernia repair with tacks and suture mesh fixation. A higher recurrence was noted in the patients who had undergone a previous open repair. The overall recurrence rate was 4.7 % during 20 months of follow-up evaluation. LeBlanc et al. [186], in a series of 200 patients (43 patients with multiple defects) reported a decreased rate of recurrence, from 9 to 4 %, when they combined tacks with suture fixation. Franklin et al. [45], in a retrospective series of 384 patients, found 11 recurrences (2.9 %) during a mean follow-up period of 47 months for patients, most of whom had mesh fixation with tacks and sutures. The findings showed that most of the recurrences (*n* = 8) occurred for patients in whom transfascial sutures were not used. Bower et al. [206] in a series of 100 patients who underwent mesh fixation with both transfascial sutures and tacks, reported a recurrence rate of 2 % during a mean follow-up period of 6.5 months. Patients with a body mass index higher than 30 kg/m² accounted for 73 % of the complications.

Perrone et al. [24] presented a series of 116 patients (28.9 % with recurrent hernias) whose hernia recurrence rate was found to be 9.3 %. In 2009, Berrevoet et al. [194] published a multicenter study of 114 patients who underwent composite mesh (Proceed) fixation with tacks and transfascial sutures. The mean recurrence rate was 3.5 % during a mean follow-up period of 27 months.

##### Studies using tacks only for mesh fixation

In a large study, Carbajo et al. [11] followed 270 patients prospectively for a median follow-up period of 44 months. Approximately 95 % of the patients had hernia defects larger than 5 cm including 147 patients with defects size 5–10 cm and 108 patients with defects larger than 10 cm. They demonstrated a recurrence rate of 4.4 %. Frantzides et al. [222] followed up 208 patients for a median of 24 months and demonstrated a recurrence rate of 1.4 % in a retrospective review. Their operative technique involved only tacks, placed 1 cm apart.

A long-term retrospective study by Bageacu et al. [107] collected data on 159 patients with a median follow-up period of 49 months. In contrast to the study by Carbajo et al. [11], this study included smaller hernia defects, with 46 % smaller than 5 cm, 24 % size 5–10 cm, and 23 % larger than 10 cm. The recurrence rate was high (15.7 %), and all recurrences were confirmed with a CT scan after clinical suspicion. The authors suggested that their higher recurrence rate might have been attributable to a technical learning curve because their recurrence rate dropped from 20 to 10 % between the periods 1993–1995 and 1996–1998, respectively.

Another study using only tacks fixation was performed by Kirshtein et al. [183] in which 103 patients were analyzed during a mean follow-up period of 26 months. They demonstrated a recurrence rate of 4 %. All four recurrences occurred within the first month, suggesting a technical cause for the failures. Gillian et al. [219] published a study of 100 patients with a mean follow-up period of 27 months. Mesh fixation was performed using a double-crown technique, and the recurrence rate was 1 %. Chowbey et al. [213] presented a series of 202 patients in whom mesh was fixed with a single crown of tacks. The recurrence rate was 1 % during a mean follow-up period of 29 months.

Wassenaar et al. [193] published a randomized controlled trial comparing mesh fixation using double-crown tacks alone, tacks with nonabsorbable sutures, and tacks with absorbable sutures and found no difference in the recurrence rate at 2 weeks, 6 weeks, and 3 months postoperatively among the three groups (*p* = 0.38, 0.76, and 0.41, respectively).

##### Studies using only transfascial suture fixation

Chelala et al. [102] analyzed 400 cases in which mesh was fixed with transfascial suture only. They also closed the hernia defect with nonabsorbable sutures. The mean operative time was 74 min, and no recurrent hernias were detected during a mean follow-up period of 28 months. Varghese et al. [181], reported that tacking of mesh to Cooper’s ligament was not sufficient. Berger et al. [182] described a case involving dislodgement of tacks when tacks alone were used to fix mesh to the pubic symphysis.

No studies have compared recurrence rates and types of mesh.

##### Comments

The current data do not give precise rates of recurrence. The total number of patients is small, and the follow-up period is short. Theoretically, laparoscopic exploration allows us to inspect the entire previous incision and to cover it with a mesh, thus reducing the probability for a recurrent hernia. On the other hand, laparoscopic repair does not always include closure of the defect and therefore often relies solely on the strength of the mesh and its fixation. More studies are required to consider these issues.

## Electronic supplementary material

Below is the link to the electronic supplementary material.

(DOCX 68 kb)

