# Supplementary material for: Guidelines for laparoscopic treatment of ventral and incisional abdominal wall hernias (International Endohernia Society [IEHS])—Part 2
Source: Surg Endosc. 2013 Nov 14;28(2):353–79. doi: 10.1007/s00464-013-3171-5 (PMC3936125; doi:10.1007/s00464-013-3171-5)
Supplement: Supplementary file 1 — (DOCX 68 kb) [file 464_2013_3171_MOESM1_ESM.docx]

List of references regarding the manuscript „ Guidelines for laparoscopic treatment of ventral and incisional abdominal wall hernias (International Endohernia Society (IEHS)) – Part 2:

Surg Endosc

DOI 10.1007/s00464-013-3171-5

**References**

1. LeBlanc KA, Booth WV. (1993)Laparoscopic repair of incisional abdominal hernias using expanded polytetrafluoroethylene: preliminary findings. Surg Laparosc Endosc. 3:39-41. **(level 4)**
2. Sauerland S, Walgenbach M, Habermalz B, Seiler CM, Miserez M. (2011)Laparoscopic versus open surgical techniques for ventral or incisional hernia repair. Cochrane Database Syst Rev. Mar 16;(3). **(level 1A)**
3. Itani KM, Kim, LT, Anthony T, Berger DH, Reda D, Neumayer L (2010)Veterans Affairs Ventral Incisional Hernia Investigators. Comparison of laparoscopic and open repair with mesh for the treatment of ventral incisional hernia: a randomized trial. Arch Surg. Apr;145(4):322-8. **(level 1B)**
4. LeBlanc KA, Elieson, MJ, Corder JM. (2007)Enterotomy and mortality rates of laparoscopic incisional and ventral hernia repair: A review of the literature. JSLS 11:408–414. **(level 1A)**
5. Ramshaw BJ, Esartia P, Schwab J, [Mason EM](http://www.ncbi.nlm.nih.gov/pubmed?term=Mason%20EM%5BAuthor%5D&cauthor=true&cauthor_uid=10484084), [Wilson RA](http://www.ncbi.nlm.nih.gov/pubmed?term=Wilson%20RA%5BAuthor%5D&cauthor=true&cauthor_uid=10484084), [Duncan TD](http://www.ncbi.nlm.nih.gov/pubmed?term=Duncan%20TD%5BAuthor%5D&cauthor=true&cauthor_uid=10484084), [Miller J](http://www.ncbi.nlm.nih.gov/pubmed?term=Miller%20J%5BAuthor%5D&cauthor=true&cauthor_uid=10484084), [Lucas GW](http://www.ncbi.nlm.nih.gov/pubmed?term=Lucas%20GW%5BAuthor%5D&cauthor=true&cauthor_uid=10484084), [Promes J](http://www.ncbi.nlm.nih.gov/pubmed?term=Promes%20J%5BAuthor%5D&cauthor=true&cauthor_uid=10484084). (1999): Comparison of laparoscopic and open ventral herniorrhaphy. Am Surg 65(9):827-831, discussion 831-2. **(level 3)**
6. Heniford BT, Park A, Ramshaw BJ, Voeller G(2003): Laparoscopic repair of ventral hernias: nine years' experience with 850 consecutive hernias. Ann Surg 238(3):391-399,discussion 399-400. **(level 4)**
7. Lederman AB, Ramshaw BJ. (2005)A Short-Term Delayed Approach to Laparoscopic Ventral Hernia When Injury Is Suspected. Surgical Innovation, 12, No 1 (March): 31-35. **(level 4)**
8. Salameh JR, Sweeney JF, Graviss EA, et al(2002): Laparoscopic ventral hernia repair during the learning curve. Hernia 6(4):182-187. **(level 4)**
9. Burger JWA, Luijendijk RW, Hop WCJ, Halm JA, Verdaasdonk EGG, Jeekel J. (2004) Long-term follow-up of a randomized controlled trial of suture versus mesh repair of incisional hernia. Annals of Surgery 240(4):578–85. **(level 1B)**
10. LeBlanc KA. (2004) Laparoscopic incisional and ventral hernia repair: Complications—how to avoid and handle. Hernia 8: 323–331. **(level 5)**
11. Carbajo MA, Martın del Olmo JC, Blanco JI, Toledano M, de la Cuesta C, Ferreras C, Vaquero C. (2003)Laparoscopic approach to incisional hernia-lessons learned from 270 patients over 8 years. Surg Endosc 17: 118–122. **(level 4)**
12. Franklin ME, Gonzalez JJ, Glass JL. (2004) Use of porcine small intestinal submucosa as a prosthetic device for laparoscopic repair of hernias in contaminated fields: 2-year follow-up. Hernia. 8(3):186-9. **(level 4)**

Moreno Egea DA, Torralba Martinez JA, Morales Cuenca G, De Miquel, Martín Lorenzo JG, AguayoAlbasini JL, CanterasJordana M. (2004) Mortality following laparoscopic ventral hernia repair: lessons from 90 consecutive cases and bibliographical analysis. Hernia 8: 208–212. **(level 3)**

Koehler RK, Voeller G. (1999)Recurrences in Laparoscopic Incisional Hernia Repairs: A Personal Series and Review of the Literature. JSLS 3:293-304. **(level4)**

Wright BE, Niskanen BD, Peterson DJ, Ney AL, Odland MD, VanCamp J, Zera RT, Rodriquez JL. (2002)Laparoscopic ventral hernia repair: are there comparative advantages over traditional methods of repair? Am Surg 68(3):291-296**.(level 3)**

Forbes SS, Eskicioglu C, McLeod RS, Okrainec A. (2009) Meta-analysis of randomized controlled trials comparing open and laparoscopic ventral and incisional hernia repair with mesh. British Journal of Surgery 96: 851–858 **(level 1A)**

Müller-Riemenschneider F, Roll S, Fredrich M, Zieren J, Reinhold T, von der Schulenburg JMG, Greiner W, Willich SN. (2007) Medical effectiveness and safety of conventional compared to laparoscopic incisional hernia repair: A systematic review. SurgEndosc 21: 2127–2136. **(level 2A)**

Carlson MA, Frantzides CT, Shostrom VK, Laguna LE. (2008) Minimally invasive ventral herniorrhaphy: an analysis of 6,266 published cases. Hernia 12:9-22**.(Level 2A)**

Barbaros U, Asoglu O, Seven R, Erbil Y, Dinccag A, Deveci U, Ozarmagan S, Mercan S. (2007)The comparison of laparoscopic and open ventral hernia repairs: a prospective randomized study. Hernia 11:51–56. **(level 1B)**

Baccari P, Nifosi J, Ghirardelli L, Staudacher C. (2009) Laparoscopic Incisional and Ventral Hernia Repair Without Sutures: A Single-Center Experience with 200 Cases. J Laparoendo Adv Surg Tech 19(2):175-179**.(Level 4)**

Ben-Haim M, Kuriansky J, Tal R, Zmora O, Mintz Y, Rosin D, Ayalon A, Shabtai M. (2002)Pitfalls and complications with laparoscopic intraperitoneal expanded polytetrafluoroethylene patch repair of postoperative ventral hernia.Lessons from the first 100 consecutive cases. SurgEndosc 16:785-788. **(Level 4)**

Berger D, Bientzle M, Müller A. (2002) Postoperative complications after laparoscopic incisional hernia repair. Incidence and treatment. SurgEndosc 16: 1720–1723**.(Level 4)**

Binenbaum SJ, Goldfarb MA. (2006) Inadvertent Enterotomy in Minimally Invasive Abdominal Surgery. JSLS 10(3):336-340**.(Level 4)**

Perrone JM, Soper NJ, Eagon C, Klingensmith ME, Aft RL, Frisella MM, Brunt M(2005) Perioperative outcomes and complications of laparoscopic ventral hernia repair. Surgery 138:708-16**.(Level 4)**

Wara P, Anderson LM. (2011) Long-term follow-up of laparoscopic repair of parastomal hernia using a bilayer mesh with a slit. Surg Endosc 25:526–530**.(Level 4)**

Serala AI. (2006) Controversies in laparoscopic repair of incisional hernia. J Minim Access Surg 2(1):7-11**.(Level 5)**

1. National Nosocomial Infections Surveillance (NNIS) report, data summary from October 1986-April 1996, issued May 1996. A report from the National Nosocomial Infections Surveillance (NNIS) System. Am J Infect Control 1998;24(5):380–8**.(level 2c)**
2. Franklin ME, Dorman JP, Glass JL, Balli JE, Gonzalez JJ.(1998) Laparoscopic Ventral and Incisional Hernia Repair. Surgical Laparoscopy Endoscopy & Percutaneous Techniques [Internet]. 8(4) :294-299 **(level 3)**
3. DenHartog D, Dur AHM, Tuinebreijer WE, Kreis RW.(2008) Open surgical procedures for incisional hernias. Cochrane Database of Systematic Reviews Issue 3. Art. No.: CD006438. **(level 1A)**
4. Zuvela M, Milićević M, Galun D, Lekić N, Basarić D, Tomić D, Petrović M, Palibrk I. .(2005) Infection in hernia surgery. Acta Chir Iugosl. 52(1):9-26.**(level 5)**
5. Kensarah AM. Dunne JR, Malone DL, Tracy JK, Napolitano LM. .(2011) A Long-term Follow-up: Suture versus Mesh Repair for Adult Umbilical Hernia in Saudi Patients. A Single Center Prospective Study. Surgical Science. 2(03):155–8**. (level 3)**
6. Chowbey PK, Sharma A, Mehrotra M, Khullar R, Soni V, Baijal M. .(2006) Laparoscopic repair of ventral / incisional hernias. J Min Access Surg 2:192-8 **(level 3)**
7. Perl TM., Golub JE. .(1998) New approaches to reduce Staphylococcus aureus nosocomial infection rates: Treating S. aureus nasal carriage. Annals of Pharmacotherapy 32(1): S7-16**.(level 4)**
8. Razavi S, Ibrahimpoor M, Sabouri Kashani A, Jafarian A. .(2005) Abdominal surgical site infections: incidence and risk factors at an Iranian teaching hospital. BMC Surgery [Internet]. 5(1):2**.(level 4)**
9. Dunne JR, Malone DL, Tracy JK, Napolitano LM. .(2003) Abdominal wall hernias: risk factors for infection and resource utilization. Journal of Surgical Research. 111(1):78–84. **(level 2c)**
10. Malone DL, Genuit T, Tracy JK, Gannon C, Napolitano LM. .(2002) Surgical site infections: reanalysis of risk factors. J Surg Res. 103(1):89-95.**(level 3)**
11. Anaya DA, Dellinger EP. .(2006) The Obese Surgical Patient. Surgical infections 7(5): 473-480 **(level 4)**
12. Kurz A, Sessler DI, Lenhardt R. .(1996) Perioperative normathermia to reduce the incidence of surgical wound infection and shorten hospitalization. New England Journal of Medicine. 334:1209–1215**.(level 1B)**
13. Cheadle WG. .(2006) Risk factors for surgical site infection. Surg Infect (Larchmt). 7 Suppl 1:S7-11 **(level 4)**
14. Mangram AJ, Horan TC, Pearson ML, Silver LC, Jarvis WR; .(1999) The Hospital Infection Control Practices Advisory Committee. Guideline for prevention of surgical site infection, 1999. Infect Control Hosp Epidemiol. 20:247-278. **(level 4)**
15. Boni L, Benevento A, Rovera F, Dionigi G, Di Giuseppe M, Bertoglio C, Dionigi R. .(2006) Infective complications in laparoscopic surgery. Surg Infect (Larchmt). 7 Suppl 2:S109-11**.(level 5)**
16. Seropian R, Reynolds BM. .(1970) Wound infection after preoperative depilation versus razor preparation. Am J Surg 12: 251-254.**(level 4)**
17. Hill GE, Frawley WH, Griffith KE, et al. .(2003) Allogeneic blood transfusion increases the risk of postoperative bacterial infection: a meta-analysis. J Trauma 54(5):908–14.**(level 1A)**
18. Eriksen JR, Gogenur T, Rosenberg J. .(2007) Choice of mesh for laparoscopic ventral hernia repair. Hernia 11:481–492**.(level 3)**
19. Franklin ME Jr, Gonzalez JJ Jr, Glass JL, Manjarrez A. .(2004) Laparoscopic ventral and incisional hernia repair: an 11-year experience. Hernia. 8(1):23-7.Epub 2003 Sep 20. **(level 4)**
20. Xourafas D, Lipsitz SR, Negro P, Ashley SW, Tavakkolizadeh A. .(2010) Impact of mesh use on morbidity following ventral hernia repair with a simultaneous bowel resection. Archives of surgery. 145(8):739–44. Available from: <http://www.ncbi.nlm.nih.gov/pubmed/20713925(level> **3)**
21. Leber GE, Garb JL, Alexander AI, Reed WD. .(2008) Long term complications associated with prosthetic repair of incisional hernias. Arch Surg. 1998;133:378–82 **(level 3)**
22. Kirby JP, Mazuski JE. .(2009) Prevention of surgical site infection. The Surgical clinics of North America. Apr;89(2):365–89,9 **(level 5)**
23. Muller  JM, Brenner  U, Dienst  C , et al. .(1982)  Preoperative parenteral feeding in patients with gastrointestinal carcinoma.  Lancet.  1:68–71**.(level 3)**
24. Latham  R, Lancaster  AD, Covington  JF , et al. .(2001) The association of diabetes and glucose control with surgicalsite infections among cardiothoracic surgery patients.  Infection Control & Hospital Epidemiology.  22:607–612.**(level 3)**
25. Yerdel  MA, Akin  EB, Dololan  S ,[Turkcapar AG](http://www.ncbi.nlm.nih.gov/pubmed?term=Turkcapar%20AG%5BAuthor%5D&cauthor=true&cauthor_uid=11141221), [Pehlivan M](http://www.ncbi.nlm.nih.gov/pubmed?term=Pehlivan%20M%5BAuthor%5D&cauthor=true&cauthor_uid=11141221), [Gecim IE](http://www.ncbi.nlm.nih.gov/pubmed?term=Gecim%20IE%5BAuthor%5D&cauthor=true&cauthor_uid=11141221), [Kuterdem E](http://www.ncbi.nlm.nih.gov/pubmed?term=Kuterdem%20E%5BAuthor%5D&cauthor=true&cauthor_uid=11141221). (2001) Effect of singledose prophylactic ampicillin and sulbactam on wound infection after tension-free inguinal hernia repair with polypropylene mesh.  Ann Surg.  233:26–33. **(level 1B)**
26. Pierce RA, Spitler JA, Frisella MM, Matthews BD, Brunt LM .(2007) Pooled data analysis of laparoscopic vs. open ventral hernia repair: 14 years of patient data accrual. Surg Endosc 21:378-386 **(level 2A)**
27. Fortelny RH, Petter-Puchner AH, Glaser KS, Offner F, Benesch T, Rohr M .(2010) Adverse effects of polyvinylidene fluoride-coated polypropylene mesh used for laparscopic intraperitoneal onlay repair of incisional hernia. British Journal of Surgery 97:1140-1145 **(level 4)**
28. Aguila B, Chapital AB, Madura JA, Harold KL .(2010) Conservative Management of Mesh-Site Infection in Hernia Repair. Journal of Laparoendoscopic & Advanced Surgical Techniques 20, Number 3, 249-253 **(level 5)**
29. Trunzo JA, Ponsky JL, Jin J. Williams CP, Rosen MJ.(2009) A novel approach for salvaging infected prosthetic mesh after ventral hernia repair. Hernia 13:545-549 **(level 5)**
30. Hawn MT, Gray SH, Snyder CW, Graham LA, Finan KR, Vick CC.(2011) Predictors of mesh explantation after incisional hernia repair. Am J Surg 202:28-33 **(level 2B)**
31. Petersen S, Henke G, Freitag M, Faulhaber A, Ludwig K.(2001) Deep Prosthesis Infection in Incisional Hernia Repair: predictive Factors and Clinical Outcome. Eur J Surg 167:453-457 **(level 3)**
32. Saettele TM, Bachmann SL, Costello CR, Grant SA, Cleveland DS, Loy TS, Kolder DG, Ramshaw BJ .(2007) Use of porcine dermal collagen as a prosthetic mesh in a contaminated field for ventral hernia repair: a case report. Hernia 11:279-285 **(level 5)**
33. Sanchez VM, Abi-Haidar YE, Itani KMF.(2011) Mesh Infection in Ventral Incisional Hernia Repair: Incidence, Contributing Factors and Treatment. Surgical Infections 12, Number 3, 205-210 **(level 5)**
34. Baharestani MM, Gabriel A .(2010) Use of negative pressure wound therapy in the management of infected abdominal wounds containing mesh: an analysis of outcomes. International Wound Journal 8, No 2, 118-125 **(level 4)**
35. Tamhankar AP, Ravi K, Everitt NJ.(2009) Vacuum Assisted Closure Therapy In The Treatment Of Mesh Infection After Hernia Repair. Surgeon 5:316-318 **(level 5)**
36. Kaafarani HM, Hur K, Hirter A, Kim LT, Thomas A, Berger DH, Reda D, Itani KM (2009) Seroma in ventral incisional herniorrhaphy: incidence, predictors and outcome. Am J Surg 198**:**639-644 **(level 1B)**
37. Tsimoyiannis EC, Siakas P, Glantzounis G, Koulas S, Mavridou P, Gossios KI (2001) Seroma in laparoscopic ventral hernioplasty. Surg Laparosc Endosc Percutan Tech 11**:**317-321 **(level 2B)**
38. Kapischke M, Schulz T, Schipper T, Tensfeldt J, Caliebe A (2008) Open versus laparoscopic incisional hernia repair: something different from a meta-analysis. Surg Endosc 22**:**2251-2260 **(level 2A)**
39. Palanivelu C, Jani KV, Senthilnathan P, Parthasarathi R, Madhankumar MV, Malladi VK (2007) Laparoscopic sutured closure with mesh reinforcement of incisional hernias. Hernia 11**:**223-228 **(level 4)**
40. Susmallian S, Gewurtz G, Ezri T, Charuzi I (2001) Seroma after laparoscopic repair of hernia with PTFE patch: is it really a complication? Hernia 5**:**139-141 **(level 4)**
41. Tsimoyiannis EC, Tsimogiannis KE, Pappas-Gogos G, Nikas K, Karfis E, Sioziou H (2008) Seroma and recurrence in laparoscopic ventral hernioplasty. JSLS 12**:**51-57 **(level 2B)**
42. Prasad P, Tantia O, Patle NM, Khanna S, Sen B (2011) Laparoscopic ventral hernia repair: a comparative study of transabdominal preperitoneal versus intraperitoneal onlay mesh repair. J Laparoendosc Adv Surg Tech A 21**:**477-483 **(level 3)**
43. Razman J, Shaharin S, Lukman MR, Sukumar N, Jasmi AY (2006) Initial experience of laparoscopic incisional hernia repair. Med J Malaysia 61**:**142-146 **(level 4)**
44. Sanchez LJ, Bencini L, Moretti R (2004) Recurrences after laparoscopic ventral hernia repair: results and critical review. Hernia 8**:**138-143 **(level 4)**
45. Toy FK, Bailey RW, Carey S, Chappuis CW, Gagner M, Josephs LG, Mangiante EC, Park AE, Pomp A, Smoot RT, Jr., Uddo JF, Jr., Voeller GR (1998) Prospective, multicenter study of laparoscopic ventral hernioplasty. Preliminary results. Surg Endosc 12**:**955-959 **(level 4)**
46. Uranues S, Salehi B, Bergamaschi R (2008) Adverse events, quality of life, and recurrence rates after laparoscopic adhesiolysis and recurrent incisional hernia mesh repair in patients with previous failed repairs. J Am Coll Surg 207**:**663-669 **(level 4)**
47. Bingener J, Buck L, Richards M, Michalek J, Schwesinger W, Sirinek K (2007) Long-term outcomes in laparoscopic vs open ventral hernia repair. Arch Surg 142**:**562-567 **(level 3)**
48. Olmi S, Erba L, Magnone S, Bertolini A, Croce E (2006) Prospective clinical study of laparoscopic treatment of incisional and ventral hernia using a composite mesh: indications, complications and results. Hernia 10**:**243-247 **(level 4)**
49. Tagaya N, Mikami H, Aoki H, Kubota K (2004) Long-term complications of laparoscopic ventral and incisional hernia repair. Surg Laparosc Endosc Percutan Tech 14**:**5-8 **(level 4)**
50. Lau H, Patil NG, Yuen WK, Lee F (2002) Laparoscopic incisional hernioplasty utilising on-lay expanded polytetrafluoroethylene DualMesh: prospective study. Hong Kong Med J 8**:**413-417 **(level 4)**
51. Birch DW (2007) Characterizing laparoscopic incisional hernia repair. Can J Surg 50**:**195-201 **(level 4)**
52. Edwards C, Angstadt J, Whipple O, Grau R (2005) Laparoscopic ventral hernia repair: postoperative antibiotics decrease incidence of seroma-related cellulitis. Am Surg 71**:**931-935; discussion 935-936 **(level 3)**
53. Eid GM, Prince JM, Mattar SG, Hamad G, Ikrammudin S, Schauer PR (2003) Medium-term follow-up confirms the safety and durability of laparoscopic ventral hernia repair with PTFE. Surgery 134**:**599-603; discussion 603-594 **(level 4)**
54. Ferranti F, Passa G, Stefanuto A, Quintiliani A (2008) Laparoscopic incisional hernia repair: our experience with 105 consecutive cases. Chir Ital 60**:**249-255 **(level 4)**
55. Parker HH, 3rd, Nottingham JM, Bynoe RP, Yost MJ (2002) Laparoscopic repair of large incisional hernias. Am Surg 68**:**530-533; discussion 533-534 **(level 4)**
56. Sharma A, Mehrotra M, Khullar R, Soni V, Baijal M, Chowbey PK (2011) Laparoscopic ventral/incisional hernia repair: a single centre experience of 1,242 patients over a period of 13 years. Hernia 15**:**131-139 **(level 3)**
57. Sodergren MH, Swift I (2010) Seroma formation and method of mesh fixation in laparoscopic ventral hernia repair--highlights of a case series. Scand J Surg 99**:**24-27 **(level 4)**
58. Yavuz N, Ipek T, As A, Kapan M, Eyuboglu E, Erguney S (2005) Laparoscopic repair of ventral and incisional hernias: our experience in 150 patients. J Laparoendosc Adv Surg Tech A 15**:**601-605 **(level 4)**
59. Jadad AR, Moore RA, Carroll D, Jenkinson C, Reynolds DJ, Gavaghan DJ, McQuay HJ (1996) Assessing the quality of reports of randomized clinical trials: is blinding necessary? Controlled clinical trials 17**:**1-12
60. Schoenmaeckers EJ, Wassenaar EB, Raymarkers JT, Rakic S(2010) Bulging of the mesh after laparoscopic repair of ventral and incisional hernias . JSLS 14 (4), 541-6 **(level 2C)**
61. Kurmann A, Visth E, Candinas D, Beldi G. (2011) Long-term follow-up of open and laparoscopic repair of large incisional hernias. World J Surg. 35(2):297-301. **(level 2B)**
62. Orenstein SB, Dumeer JL, Montegudo J, Poi MJ, Novitsky YW(2011) Outcome of laparoscopic ventral hernia repair with routine defect closure using “shoelacing” technique. Surg Endosc, 25 (5):1452-7 **(level 4)**
63. Aasvang E KH (1986) Classification of chronic pain. Descriptions of chronic pain syndromes and definitions of pain terms. Prepared by the International Association for the Study of Pain. SUbcommittee on Taxonomy. Oaub Syook 3**:**1-226
64. Kehlet H, Rathmell JP (2010) Persistent postsurgical pain: the path forward through better design of clinical studies. Anesthesiology 112**:**514-515 **(level 5)**
65. Khan RS, Ahmed K, Blakeway E, Skapinakis P, Nihoyannopoulos L, Macleod K, Sevdalis N, Ashrafian H, Platt M, Darzi A, Athanasiou T (2011) Catastrophizing: a predictive factor for postoperative pain. Am J Surg 201**:**122-131 **(level 2A)**
66. Sajid MS, Bokhari SA, Mallick AS, Cheek E, Baig MK (2009) Laparoscopic versus open repair of incisional/ventral hernia: a meta-analysis. Am J Surg 197**:**64-72 **(level 1A)**
67. Wassenaar E, Schoenmaeckers E, Raymakers J, van der Palen J, Rakic S (2010) Mesh-fixation method and pain and quality of life after laparoscopic ventral or incisional hernia repair: a randomized trial of three fixation techniques. Surg Endosc 24**:**1296-1302 **(level 2B)**
68. Snyder CW, Graham LA, Vick CC, Gray SH, Finan KR, Hawn MT (2011) Patient satisfaction, chronic pain, and quality of life after elective incisional hernia repair: effects of recurrence and repair technique. Hernia 15**:**123-129 **(level 2C)**
69. Rosen MJ, Duperier T, Marks J, Onders R, Hardacre J, Ponsky J, Ermlich B, Laughinghouse M (2009) Prospective randomized double-blind placebo-controlled trial of postoperative elastomeric pain pump devices used after laparoscopic ventral hernia repair. Surg Endosc 23**:**2637-2643 **(level 1B)**
70. Nguyen SQ, Divino CM, Buch KE, Schnur J, Weber KJ, Katz LB, Reiner MA, Aldoroty RA, Herron DM (2008) Postoperative pain after laparoscopic ventral hernia repair: a prospective comparison of sutures versus tacks. JSLS 12**:**113-116 **(level 2B)**
71. Moreno-Egea A, Carrillo A, Aguayo JL (2008) Midline versus nonmidline laparoscopic incisional hernioplasty: a comparative study. Surg Endosc 22**:**744-749 **(level 3)**
72. Lepere M, Benchetrit S, Bertrand JC, Chalbet JY, Combier JP, Detruit B, Herbault G, Jarsaillon P, Lagoutte J, Levard H, Rignier P (2008) Laparoscopic resorbable mesh fixation. Assessment of an innovative disposable instrument delivering resorbable fixation devices: I-Clip(TM). Final results of a prospective multicentre clinical trial. Hernia 12**:**177-183 **(level 4)**
73. Johanet H, Dabrowski A, Hauters P (2006) Laparoscopic cure of small ventral hernias with composite mesh. Hernia 10**:**414-418 **(level 4)**
74. Hope WW, Lincourt AE, Newcomb WL, Schmelzer TM, Kercher KW, Heniford BT (2008) Comparing quality-of-life outcomes in symptomatic patients undergoing laparoscopic or open ventral hernia repair. J Laparoendosc Adv Surg Tech A 18**:**567-571 **(level 3)**
75. Eriksen JR, Poornoroozy P, Jorgensen LN, Jacobsen B, Friis-Andersen HU, Rosenberg J (2009) Pain, quality of life and recovery after laparoscopic ventral hernia repair. Hernia 13**:**13-21 **(level 4)**
76. Chelala E, Thoma M, Tatete B, Lemye AC, Dessily M, Alle JL (2007) The suturing concept for laparoscopic mesh fixation in ventral and incisional hernia repair: Mid-term analysis of 400 cases. Surg Endosc 21**:**391-395 **(level 4)**
77. Carbonell AM, Harold KL, Mahmutovic AJ, Hassan R, Matthews BD, Kercher KW, Sing RF, Heniford BT (2003) Local injection for the treatment of suture site pain after laparoscopic ventral hernia repair. Am Surg 69**:**688-691; discussion 691-682 **(level 4)**
78. Bellows CF, Berger DH (2006) Infiltration of suture sites with local anesthesia for management of pain following laparoscopic ventral hernia repairs: a prospective randomized trial. JSLS 10**:**345-350 **(level 2B)**
79. Beldi G, Wagner M, Bruegger LE, Kurmann A, Candinas D (2011) Mesh shrinkage and pain in laparoscopic ventral hernia repair: a randomized clinical trial comparing suture versus tack mesh fixation. Surg Endosc 25**:**749-755 **(level 2B)**
80. Bansal VK, Misra MC, Kumar S, Rao YK, Singhal P, Goswami A, Guleria S, Arora MK, Chabra A (2011) A prospective randomized study comparing suture mesh fixation versus tacker mesh fixation for laparoscopic repair of incisional and ventral hernias. Surg Endosc 25**:**1431-1438 **(level 2B)**
81. Bageacu S, Blanc P, Breton C, Gonzales M, Porcheron J, Chabert M, Balique JG (2002) Laparoscopic repair of incisional hernia: a retrospective study of 159 patients. Surg Endosc 16**:**345-348 **(level 4)**
82. Wolter A, Rudroff C, Sauerland S, Heiss MM (2009) Laparoscopic incisional hernia repair: evaluation of effectiveness and experiences. Hernia 13**:**469-474 **(level 3)**
83. Pring CM, Tran V, O'Rourke N, Martin IJ (2008) Laparoscopic versus open ventral hernia repair: a randomized controlled trial. ANZ J Surg 78**:**903-906 **(level 1B)**
84. Moreno-Egea A, Bustos JA, Girela E, Aguayo-Albasini JL (2010) Long-term results of laparoscopic repair of incisional hernias using an intraperitoneal composite mesh. Surg Endosc 24**:**359-365 **(level4)**
85. Misiakos EP, Machairas A, Patapis P, Liakakos T (2008) Laparoscopic ventral hernia repair: pros and cons compared with open hernia repair. JSLS 12**:**117-125 **(level 5)**
86. Misra MC, Bansal VK, Kulkarni MP, Pawar DK (2006) Comparison of laparoscopic and open repair of incisional and primary ventral hernia: results of a prospective randomized study. Surg Endosc 20**:**1839-1845 **(level 2B)**
87. Lomanto D, Iyer SG, Shabbir A, Cheah WK (2006) Laparoscopic versus open ventral hernia mesh repair: a prospective study. Surg Endosc 20**:**1030-1035 **(level 2B)**
88. Levard H, Curt F, Perniceni T, Denet C, Gayet B (2006) [Laparoscopic incisional hernia repair: prospective non randomized trial in 51 cases]. Ann Chir 131**:**244-249 **(level 4)**
89. Antinori A, Moschella F, Maci E, Accetta C, Nunziata J, Magistrelli P (2008) [Immediate and long-term results after laparoscopic primary ventral hernia repair]. Ann Ital Chir 79**:**435-439 **(level 4)**
90. Asencio F, Aguilo J, Peiro S, Carbo J, Ferri R, Caro F, Ahmad M (2009) Open randomized clinical trial of laparoscopic versus open incisional hernia repair. Surg Endosc 23**:**1441-1448 **(level 1B)**
91. Bencini L, Sanchez LJ, Scatizzi M, Farsi M, Boffi B, Moretti R (2003) Laparoscopic treatment of ventral hernias: prospective evaluation. Surg Laparosc Endosc Percutan Tech 13**:**16-19 **(level 4)**
92. Olmi S, Cesana G, Sagutti L, Pagano C, Vittoria G, Croce E (2010) Laparoscopic incisional hernia repair with fibrin glue in select patients. JSLS : Journal of the Society of Laparoendoscopic Surgeons / Society of Laparoendoscopic Surgeons 14**:**240-245 **(level 4)**
93. Olmi S, Scaini A, Erba L, Croce E (2007) Use of fibrin glue (Tissucol) in laparoscopic repair of abdominal wall defects: preliminary experience. Surg Endosc 21**:**409-413 **(level 4)**
94. Brill JB, Turner PL (2011) Long-term outcomes with transfascial sutures versus tacks in laparoscopic ventral hernia repair: a review. Am Surg 77**:**458-465 **(level 2A)**
95. Navarra G, Musolino C, De Marco ML, Bartolotta M, Barbera A, Centorrino T (2007) Retromuscular sutured incisional hernia repair: a randomized controlled trial to compare open and laparoscopic approach. Surg Laparosc Endosc Percutan Tech 17**:**86-90 **(level 1B)**
96. Wright D, Paterson C, Scott N, Hair A, O'Dwyer PJ (2002) Five-year follow-up of patients undergoing laparoscopic or open groin hernia repair: a randomized controlled trial. Annals of surgery 235**:**333-337 **(level 1B)**
97. Dennis R, O'Riordan D (2007) Risk factors for chronic pain after inguinal hernia repair. Annals of the Royal College of Surgeons of England 89**:**218-220 **(level 3B)**
98. Courtney C, Duffy K, Serpell M, O'Dwyer P (2002) Outcome of patients with chronic pain following repair of groin hernia. British Journal of Surgery 89**:**1310-1314 **(level 3B)**
99. Poobalan AS, Bruce J, Smith WC, King PM, Krukowski ZH, Chambers WA (2003) A review of chronic pain after inguinal herniorrhaphy. The Clinical journal of pain 19**:**48-54 **(level 1A)**
100. Berndsen F, Petersson U, Arvidsson D, Leijonmarck C-E, Rudberg C, Smedberg S, Montgomery A (2008) Discomfort five years after laparoscopic and Shouldice inguinal hernia repair: a randomised trial with 867 patients. A report from the SMIL study group. Hernia 11**:**307-313 **(level 1B)**
101. Callesen T, Bech K, Kehlet H (1999) Prospective study of chronic pain after groin hernia repair. The British journal of surgery 86**:**1528-1531 **(level 1B)**
102. Poobalan AS, Bruce J, King PM, Chambers WA, Krukowski ZH, Smith WC (2001) Chronic pain and quality of life following open inguinal hernia repair. The British journal of surgery 88**:**1122-1126
103. Chowbey PK, Sharma A, Mehrotra M, Khullar R, Soni V, Baijal M.(2006) Laparoscopic repair of ventral / incisional hernias. J Min Access Surg 2:192-8 **(level 3)**
104. Klinge U, Conze J, Krones CJ, Schumpelik V.(2005) Incisional hernia: Open techniques. World J Surg 29:1066-72**.(level 5)**
105. Bencini L, Sanchez LJ, Bernini M, Miranda E, Farsi M, Boffi B, Moretti R. (2009) Predictors of recurrence after laparoscopic ventral hernia repair. Surg Laparosc Endosc Percutan Tech. 19(2):128-32**.(level 3)**
106. Rosen M, Brody F, Ponsky J, Walsh RM, Rosenblatt S, Duperier F, Fanning A, Siperstein A. (2003) Recurrence after laparoscopic ventral hernia repair. Surg Endosc. 17(1):123-8. Epub 2002 Sep 23**. (level 3)**
107. Paul A, Korenkov M, Peters S, Kohler L, Fischer S, Troidl H. (1998) Unacceptable results of the Mayo procedure for repair of abdominal incisional hernias. Eur J Surg. 164:361-367. **(level 3)**
108. Burger JW, Luijendijk RW, Hop WC, Halm JA, Verdaasdonk EG, Jeekel J. (2004) Long-term follow-up of a randomized controlled trial of suture versus mesh repair of incisional hernia. Ann Surg. 240:578-583**.(level 1B)**
109. Wassenaar EB, Schoenmaeckers EJ, Raymakers JT, Rakic S. (2009) Recurrences after laparoscopic repair of ventral and incisional hernia: lessons learned from 505 repairs. Surg Endosc. 23(4):825-32. Epub 2008 Sep 24**.(level 4)**
110. Biondi A, Tropea A, Monaco G, Musmeci N, Zanghi G, Basile F. (2010) Complications in the laparoscopic treatment of primary and secondary hernias of the abdominal wall. Ann Ital Chir. 81(3):193-8**. (level 3)**
111. P K Chowbey et al, (2006) Max institute of MAMBS, India SYMPOSIUM. 2(3):192-98 **(level 3)**
112. Barzana D, Johnson K, Clancy TV, Hope WW. (2012) Hernia recurrence through a composite mesh secondary to transfascial suture holes. Hernia. 16(2):219-21. Epub 2010 Sep 12.**(level 4)**
113. Liang MK, Clapp ML, Garcia A, Subramanian A, Awad SS. (2012) Mesh shift following laparoscopic ventral hernia repair. J Surg Res. 177: 7-13**(4)**
114. Muysoms F, Daeter E, Mijnsbrugge GV, Claeys D. (2004) Laparoscopic intraperitoneal repair of incisional and ventral hernias. Acta Chirurgica Belgica. 104(6): 705-708 **(level 4)**
115. LeBlanc KA, Whitaker JM, Bellanger DE, Rhynes VK. (2003) Laparoscopic incisional and ventral hernioplasty : lessons learned from 200 patients. Hernia. 7(3):118-24. Epub 2003 Mar 21**.(level 3)**
116. Sharma A, Dey A, Khullar R, Soni V, Baijal M, Chowbey PK. (2011) Laparoscopic repair of suprapubic hernias: transabdominal partial extraperitoneal (TAPE) technique. Surg Endosc. 25(7):2147-52. Epub 2010 Dec 24**.(level 3)**
117. Olmi S, Scaini A, Cesana GC, Erba L, Croce E. (2007) Laparoscopic versus open incisional hernia repair: an open randomized controlled study. Surgical endoscopy 21(4):555-9. **(1B)**
118. Moreno-Egea A, Carrasco L, Girela E, Martin JG, Aguayo JL, Canteras M. (2002) Open vs laparoscopic repair of spigelian hernia: a prospective randomized trial. Archives of surgery (Chicago, Ill. : 1960) 137(11):1266-8. **(1B)**
119. Carbajo MA, Martin del Olmo JC, Blanco JI, de la Cuesta C, Toledano M, Martin F, et al. (1999) Laparoscopic treatment vs open surgery in the solution of major incisional and abdominal wall hernias with mesh. Surg Endosc 13(3):250-2. **(1B)**
120. Pham CT, Perera CL, Watkin DS, Maddern GJ. (2009) Laparoscopic ventral hernia repair: a systematic review. Surgical endoscopy 23(1):4-15. **(1A)**
121. Goodney PP, Birkmeyer CM, Birkmeyer JD. (2002) Short-term outcomes of laparoscopic and open ventral hernia repair: a meta-analysis. Archives of surgery (Chicago, Ill. : 1960) 137(10):1161-5. **(1A)**
122. Beldi G, Ipaktchi R, Wagner M, Gloor B, Candinas D. (2006) Laparoscopic ventral hernia repair is safe and cost effective. Surgical endoscopy 20(1):92-5. **(2B)**
123. McGreevy JM, Goodney PP, Birkmeyer CM, Finlayson SR, Laycock WS, Birkmeyer JD. (2003) A prospective study comparing the complication rates between laparoscopic and open ventral hernia repairs. Surgical endoscopy 17(11):1778-80. **(2B)**
124. Raftopoulos I, Vanuno D, Khorsand J, Kouraklis G, Lasky P. (2003) Comparison of open and laparoscopic prosthetic repair of large ventral hernias. JSLS : Journal of the Society of Laparoendoscopic Surgeons / Society of Laparoendoscopic Surgeons 7(3):227-32. **(2B)**
125. Hwang CS, Wichterman KA, Alfrey EJ. (2009) Laparoscopic ventral hernia repair is safer than open repair: analysis of the NSQIP data. The Journal of surgical research 156(2):213-6. **(2C)**
126. Sains PS, Tilney HS, Purkayastha S, Darzi AW, Athanasiou T, Tekkis PP, et al. (2006) Outcomes following laparoscopic versus open repair of incisional hernia. World journal of surgery 30(11):2056-64. **(1A)**
127. Earle D, Seymour N, Fellinger E, Perez A. (2006) Laparoscopic versus open incisional hernia repair: a single-institution analysis of hospital resource utilization for 884 consecutive cases. Surgical endoscopy 20(1):71-5. **(3)**
128. Olmi S, Magnone S, Erba L, Bertolini A, Croce E. (2005) Results of laparoscopic versus open abdominal and incisional hernia repair. JSLS : Journal of the Society of Laparoendoscopic Surgeons / Society of Laparoendoscopic Surgeons 9(2):189-95. **(3)**
129. Bencini L, Sanchez LJ, Boffi B, Farsi M, Scatizzi M, Moretti R. (2003) Incisional hernia: repair retrospective comparison of laparoscopic and open techniques. Surgical endoscopy 17(10):1546-51. **(3)**
130. Gonzalez R, Mason E, Duncan T, Wilson R, Ramshaw BJ. (2003) Laparoscopic versus open umbilical hernia repair. JSLS : Journal of the Society of Laparoendoscopic Surgeons / Society of Laparoendoscopic Surgeons 7(4):323-8. **(3)**
131. Park A, Birch DW, Lovrics P. (1998) Laparoscopic and open incisional hernia repair: a comparison study. Surgery 124(4):816-21; discussion 21-2. **(3)**
132. Bisgaard T, Kehlet H, Bay-Nielsen MB, Iversen MG, Wara P, Rosenberg J, et al. (2009) Nationwide study of early outcomes after incisional hernia repair. The British journal of surgery 96(12):1452-7. **(2C)**
133. Kaafarani HM, Kaufman D, Reda D, Itani KM. (2010) Predictors of surgical site infection in laparoscopic and open ventral incisional herniorrhaphy. The Journal of surgical research 163(2):229-34. **(1B)**
134. Zerey M, Heniford BT. (2006) Laparoscopic versus open surgery for ventral hernia repair--which is best? Nature clinical practice. Gastroenterology & hepatology 3(7):372-3. **(2B)**
135. Solomon TA, Wignesvaran P, Chaudry MA, Tutton MG. (2010) A retrospective audit comparing outcomes of open versus laparoscopic repair of umbilical/paraumbilical herniae. Surgical endoscopy 24(12):3109-12. **(3)**
136. Ballem N, Parikh R, Berber E, Siperstein A. (2008) Laparoscopic versus open ventral hernia repairs: 5 year recurrence rates. Surgical endoscopy 22(9):1935-40. **(3)**
137. van't RM, Vrijland WW, Lange JF, Hop WC, Jeekel J, Bonjer HJ. (2002) Mesh repair of incisional hernia: comparison of laparoscopic and open repair. The European journal of surgery = Acta chirurgica 168(12):684-9. **(3)**
138. Morales-Conde S. (2012) A new classification for seroma after laparoscopic ventral hernia repair. Hernia 16(3):261-7. **(5)**
139. Bansal VK, Misra MC, Babu D, Singhal P, Rao K, Sagar R, Kumar S, Rajeshwari S, Rewari V (2012) Comparison of long-term outcome and quality of life after laparoscopic repair of incisional and ventral hernias with suture fixation with and without tacks: a prospective, randomized, controlled study. Surg Endosc. 2012, Dec; 26(12): 3476-85.**(1b)**
140. Moreno-Egea A, Carrasco L, Girela E, Martín JG, Aguayo JL, Canteras M (2002) Open vs laparoscopic repair of spigelian hernia: a prospective randomized trial. Arch Surg137: 1266–8. **(1b)**
141. [DeMaria EJ](http://www.ncbi.nlm.nih.gov/pubmed?term=%22DeMaria%20EJ%22%5BAuthor%5D), [Moss JM](http://www.ncbi.nlm.nih.gov/pubmed?term=%22Moss%20JM%22%5BAuthor%5D), [Sugerman HJ](http://www.ncbi.nlm.nih.gov/pubmed?term=%22Sugerman%20HJ%22%5BAuthor%5D) (2000)Laparoscopic intraperitoneal polytetrafluoroethylene (PTFE) prosthetic patch repair of ventral hernia. Prospective comparison to open prefascial polypropylene mesh repair.[SurgEndosc](http://www.ncbi.nlm.nih.gov/pubmed?term=Demaria%20laparoscopic%20ventral%20hernia%202000)14: 326-9. **(2b)**
142. Holzman MD, Purut CM, Reintgen K, Eubanks S, Pappas TN (1997)[Laparoscopic ventral and incisional hernioplasty.](http://www.ncbi.nlm.nih.gov/pubmed/8994985) Surg Endosc 11:32-5. **(2b)**
143. [Chari R](http://www.ncbi.nlm.nih.gov/pubmed?term=%22Chari%20R%22%5BAuthor%5D), [Chari V](http://www.ncbi.nlm.nih.gov/pubmed?term=%22Chari%20V%22%5BAuthor%5D), [Eisenstat M](http://www.ncbi.nlm.nih.gov/pubmed?term=%22Eisenstat%20M%22%5BAuthor%5D), [Chung R](http://www.ncbi.nlm.nih.gov/pubmed?term=%22Chung%20R%22%5BAuthor%5D) (2000)A case controlled study of laparoscopic incisional hernia repair.[SurgEndosc](http://www.ncbi.nlm.nih.gov/pubmed?term=chari%20laparoscopic%20ventral%20hernia%202000) 14:117-9. **(3)**
144. [Zanghì A](http://www.ncbi.nlm.nih.gov/pubmed?term=Zangh%C3%AC%20A%5BAuthor%5D&cauthor=true&cauthor_uid=11347318), [Di Vita M](http://www.ncbi.nlm.nih.gov/pubmed?term=Di%20Vita%20M%5BAuthor%5D&cauthor=true&cauthor_uid=11347318), [Lomenzo E](http://www.ncbi.nlm.nih.gov/pubmed?term=Lomenzo%20E%5BAuthor%5D&cauthor=true&cauthor_uid=11347318), [De Luca A](http://www.ncbi.nlm.nih.gov/pubmed?term=De%20Luca%20A%5BAuthor%5D&cauthor=true&cauthor_uid=11347318), [Cappellani A](http://www.ncbi.nlm.nih.gov/pubmed?term=Cappellani%20A%5BAuthor%5D&cauthor=true&cauthor_uid=11347318) (2000)Laparoscopic repair vs open surgery for incisional hernias: a comparison study. [Ann Ital Chir](http://www.ncbi.nlm.nih.gov/pubmed/11347318) 71: 663-7. **(3)**
145. [Zografos GN](http://www.ncbi.nlm.nih.gov/pubmed?term=%22Zografos%20GN%22%5BAuthor%5D), [Mitropapas G](http://www.ncbi.nlm.nih.gov/pubmed?term=%22Mitropapas%20G%22%5BAuthor%5D), [Vasiliadis G](http://www.ncbi.nlm.nih.gov/pubmed?term=%22Vasiliadis%20G%22%5BAuthor%5D), [Farfaras A](http://www.ncbi.nlm.nih.gov/pubmed?term=%22Farfaras%20A%22%5BAuthor%5D), [Ageli C](http://www.ncbi.nlm.nih.gov/pubmed?term=%22Ageli%20C%22%5BAuthor%5D), [Margaris E](http://www.ncbi.nlm.nih.gov/pubmed?term=%22Margaris%20E%22%5BAuthor%5D), [Tsipras I](http://www.ncbi.nlm.nih.gov/pubmed?term=%22Tsipras%20I%22%5BAuthor%5D), [Koliopanos A](http://www.ncbi.nlm.nih.gov/pubmed?term=%22Koliopanos%20A%22%5BAuthor%5D), [Pateras J](http://www.ncbi.nlm.nih.gov/pubmed?term=%22Pateras%20J%22%5BAuthor%5D), [Papastratis G](http://www.ncbi.nlm.nih.gov/pubmed?term=%22Papastratis%20G%22%5BAuthor%5D) (2007)Open and laparoscopic approach in incisional hernia repair with ePTFE prosthesis.[J Laparoendosc Adv Surg Tech A](http://www.ncbi.nlm.nih.gov/pubmed?term=zografos%20laparoscopic%20incisional%20hernia%20repair)  17: 277-81. **(3)**
146. [Brown RB](http://www.ncbi.nlm.nih.gov/pubmed?term=Brown%20RB%5BAuthor%5D&cauthor=true&cauthor_uid=8180760).Laparoscopic hernia repair: a rural perspective (1994) [Surg Laparosc Endosc](http://www.ncbi.nlm.nih.gov/pubmed?term=brown%20rb%20incisional%20hernia) 4: 106-9. **(4)**
147. Franklin ME Jr, Dorman JP, Glass JL, Balli JE, Gonzalez JJ Jr (1998) Laparoscopic ventral and incisional hernia repair.Surg Laparosc Endosc 8: 294 – 9. **(4)**
148. [Tsimoyiannis EC](http://www.ncbi.nlm.nih.gov/pubmed?term=Tsimoyiannis%20EC%5BAuthor%5D&cauthor=true&cauthor_uid=9799145), [Tassis A](http://www.ncbi.nlm.nih.gov/pubmed?term=Tassis%20A%5BAuthor%5D&cauthor=true&cauthor_uid=9799145), [Glantzounis G](http://www.ncbi.nlm.nih.gov/pubmed?term=Glantzounis%20G%5BAuthor%5D&cauthor=true&cauthor_uid=9799145), [Jabarin M](http://www.ncbi.nlm.nih.gov/pubmed?term=Jabarin%20M%5BAuthor%5D&cauthor=true&cauthor_uid=9799145), [Siakas P](http://www.ncbi.nlm.nih.gov/pubmed?term=Siakas%20P%5BAuthor%5D&cauthor=true&cauthor_uid=9799145), [Tzourou H](http://www.ncbi.nlm.nih.gov/pubmed?term=Tzourou%20H%5BAuthor%5D&cauthor=true&cauthor_uid=9799145) (1998) Laparoscopic intraperitonealonlay mesh repair of incisional hernia.[Surg Laparosc Endosc](http://www.ncbi.nlm.nih.gov/pubmed/9799145) 8: 360-2. **(4)**
149. [Sanders LM](http://www.ncbi.nlm.nih.gov/pubmed?term=Sanders%20LM%5BAuthor%5D&cauthor=true&cauthor_uid=10219859), [Flint LM](http://www.ncbi.nlm.nih.gov/pubmed?term=Flint%20LM%5BAuthor%5D&cauthor=true&cauthor_uid=10219859), [Ferrara JJ](http://www.ncbi.nlm.nih.gov/pubmed?term=Ferrara%20JJ%5BAuthor%5D&cauthor=true&cauthor_uid=10219859) (1999) Initial experience with laparoscopic repair of incisional hernias.[Am J Surg](http://www.ncbi.nlm.nih.gov/pubmed/10219859) 177: 227-31. **(4)**
150. [Heniford BT](http://www.ncbi.nlm.nih.gov/pubmed?term=%22Heniford%20BT%22%5BAuthor%5D), [Ramshaw BJ](http://www.ncbi.nlm.nih.gov/pubmed?term=%22Ramshaw%20BJ%22%5BAuthor%5D) (2000)Laparoscopic ventral hernia repair: a report of 100 consecutive cases.[Surg Endosc](http://www.ncbi.nlm.nih.gov/pubmed/10858463)14:419-23. **(4)**
151. [LeBlanc KA](http://www.ncbi.nlm.nih.gov/pubmed?term=LeBlanc%20KA%5BAuthor%5D&cauthor=true&cauthor_uid=11387722), [Booth WV](http://www.ncbi.nlm.nih.gov/pubmed?term=Booth%20WV%5BAuthor%5D&cauthor=true&cauthor_uid=11387722), [Whitaker JM](http://www.ncbi.nlm.nih.gov/pubmed?term=Whitaker%20JM%5BAuthor%5D&cauthor=true&cauthor_uid=11387722), [Bellanger DE](http://www.ncbi.nlm.nih.gov/pubmed?term=Bellanger%20DE%5BAuthor%5D&cauthor=true&cauthor_uid=11387722) (2001) Laparoscopic incisional and ventral herniorraphy: our initial 100 patients. [Hernia](http://www.ncbi.nlm.nih.gov/pubmed/11387722) 5: 41-5. **(4)**
152. Moreno-Egea A, Castillo J, Girela E, Canteras M, Aguayo JL (2002) Outpatient laparoscopic incisional/ ventral hernioplasty: our experience in 55 cases. SurgLaparoscEndoscPercutan Tech 12:171-174. **(4)**
153. [Kua KB](http://www.ncbi.nlm.nih.gov/pubmed?term=%22Kua%20KB%22%5BAuthor%5D), [Coleman M](http://www.ncbi.nlm.nih.gov/pubmed?term=%22Coleman%20M%22%5BAuthor%5D), [Martin I](http://www.ncbi.nlm.nih.gov/pubmed?term=%22Martin%20I%22%5BAuthor%5D), [O'Rourke N](http://www.ncbi.nlm.nih.gov/pubmed?term=%22O'Rourke%20N%22%5BAuthor%5D) (2002) Laparoscopic repair of ventral incisional hernia.[ANZ J Surg](http://www.ncbi.nlm.nih.gov/pubmed?term=kua%20laparoscopic%20ventral%20hernia) 72:296-9. **(4)**
154. [Raftopoulos I](http://www.ncbi.nlm.nih.gov/pubmed?term=Raftopoulos%20I%5BAuthor%5D&cauthor=true&cauthor_uid=12590723), [Vanuno D](http://www.ncbi.nlm.nih.gov/pubmed?term=Vanuno%20D%5BAuthor%5D&cauthor=true&cauthor_uid=12590723), [Khorsand J](http://www.ncbi.nlm.nih.gov/pubmed?term=Khorsand%20J%5BAuthor%5D&cauthor=true&cauthor_uid=12590723), [Ninos J](http://www.ncbi.nlm.nih.gov/pubmed?term=Ninos%20J%5BAuthor%5D&cauthor=true&cauthor_uid=12590723), [Kouraklis G](http://www.ncbi.nlm.nih.gov/pubmed?term=Kouraklis%20G%5BAuthor%5D&cauthor=true&cauthor_uid=12590723), [Lasky P](http://www.ncbi.nlm.nih.gov/pubmed?term=Lasky%20P%5BAuthor%5D&cauthor=true&cauthor_uid=12590723) (2002) Outcome of laparoscopic ventral hernia repair in correlation with obesity, type of hernia, and hernia size.[J Laparoendosc Adv Surg Tech A](http://www.ncbi.nlm.nih.gov/pubmed/12590723) 12: 425-9. **(4)**
155. [Varghese TK](http://www.ncbi.nlm.nih.gov/pubmed?term=Varghese%20TK%5BAuthor%5D&cauthor=true&cauthor_uid=12121696), [Denham DW](http://www.ncbi.nlm.nih.gov/pubmed?term=Denham%20DW%5BAuthor%5D&cauthor=true&cauthor_uid=12121696), [Dawes LG](http://www.ncbi.nlm.nih.gov/pubmed?term=Dawes%20LG%5BAuthor%5D&cauthor=true&cauthor_uid=12121696), [Murayama KM](http://www.ncbi.nlm.nih.gov/pubmed?term=Murayama%20KM%5BAuthor%5D&cauthor=true&cauthor_uid=12121696), [Prystowsky JB](http://www.ncbi.nlm.nih.gov/pubmed?term=Prystowsky%20JB%5BAuthor%5D&cauthor=true&cauthor_uid=12121696), [Joehl RJ](http://www.ncbi.nlm.nih.gov/pubmed?term=Joehl%20RJ%5BAuthor%5D&cauthor=true&cauthor_uid=12121696) (2002) Laparoscopic ventral hernia repair: an initial institutional experience.[J Surg Res](http://www.ncbi.nlm.nih.gov/pubmed/12121696) 105: 115-8. **(4)**
156. Berger D, Bientzle M, Muller A (2002) Postoperative complications after laparoscopic incisional hernia repair. Incidence and treatment. SurgEndosc 16: 1720–1723(4)
157. Kirshtein B, Lantsberg L, Avinoach E, Bayme M, Mizrahi S (2002) Laparoscopic repair of large incisional hernias. SurgEndosc 16: 1717–1719.
158. [Aura T](http://www.ncbi.nlm.nih.gov/pubmed?term=%22Aura%20T%22%5BAuthor%5D), [Habib E](http://www.ncbi.nlm.nih.gov/pubmed?term=%22Habib%20E%22%5BAuthor%5D), [Mekkaoui M](http://www.ncbi.nlm.nih.gov/pubmed?term=%22Mekkaoui%20M%22%5BAuthor%5D), [Brassier D](http://www.ncbi.nlm.nih.gov/pubmed?term=%22Brassier%20D%22%5BAuthor%5D), [Elhadad A](http://www.ncbi.nlm.nih.gov/pubmed?term=%22Elhadad%20A%22%5BAuthor%5D) (2002)Laparoscopic tension-free repair of anterior abdominal wall incisional and ventral hernias with an intraperitoneal Gore-Tex mesh: prospective study and review of the literature. [J Laparoendosc Adv Surg Tech A](http://www.ncbi.nlm.nih.gov/pubmed/12269494) 12:263-7**. (4)**
159. [Chowbey PK](http://www.ncbi.nlm.nih.gov/pubmed?term=Chowbey%20PK%5BAuthor%5D&cauthor=true&cauthor_uid=12709615), [Sharma A](http://www.ncbi.nlm.nih.gov/pubmed?term=Sharma%20A%5BAuthor%5D&cauthor=true&cauthor_uid=12709615), [Khullar R](http://www.ncbi.nlm.nih.gov/pubmed?term=Khullar%20R%5BAuthor%5D&cauthor=true&cauthor_uid=12709615), [Soni V](http://www.ncbi.nlm.nih.gov/pubmed?term=Soni%20V%5BAuthor%5D&cauthor=true&cauthor_uid=12709615), [Baijal M](http://www.ncbi.nlm.nih.gov/pubmed?term=Baijal%20M%5BAuthor%5D&cauthor=true&cauthor_uid=12709615) (2003) Laparoscopic ventral hernia repair with extraperitoneal mesh: surgical technique and early results.[SurgLaparoscEndoscPercutan Tech](http://www.ncbi.nlm.nih.gov/pubmed?term=Chowbey%202003%20incisional%20hernia) 13: 101-5. **(4)**
160. LeBlanc KA, Whitaker JM, Bellanger DE, Rhynes VK (2003) Laparoscopic incisional and ventral hernioplasty: lessons learned from 200 patients. Hernia 7: 118–124. **(4)**
161. Heniford BT, Park A, Ramshaw BJ, Voeller G (2000) Laparoscopic ventral and incisional hernia repair in 407 patients. J Am CollSurg 190:645-50. **(4)**
162. [Tagaya N](http://www.ncbi.nlm.nih.gov/pubmed?term=Tagaya%20N%5BAuthor%5D&cauthor=true&cauthor_uid=15259577), [Mikami H](http://www.ncbi.nlm.nih.gov/pubmed?term=Mikami%20H%5BAuthor%5D&cauthor=true&cauthor_uid=15259577), [Aoki H](http://www.ncbi.nlm.nih.gov/pubmed?term=Aoki%20H%5BAuthor%5D&cauthor=true&cauthor_uid=15259577), [Kubota K](http://www.ncbi.nlm.nih.gov/pubmed?term=Kubota%20K%5BAuthor%5D&cauthor=true&cauthor_uid=15259577)(2004)Long-term complications of laparoscopic ventral and incisional hernia repair. [SurgLaparoscEndoscPercutan Tech](http://www.ncbi.nlm.nih.gov/pubmed/15259577) 14: 5-8. **(4)**
163. Ujiki MB, Weinberger J, Varghese TK, Murayama KM, Joehl RJ (2004) One hundred consecutive laparoscopic ventral hernia repairs. Am J Surg 188: 593–597. **(4)**
164. [Bamehriz F](http://www.ncbi.nlm.nih.gov/pubmed?term=Bamehriz%20F%5BAuthor%5D&cauthor=true&cauthor_uid=15472549), [Birch DW](http://www.ncbi.nlm.nih.gov/pubmed?term=Birch%20DW%5BAuthor%5D&cauthor=true&cauthor_uid=15472549)(2004)The feasibility of adopting laparoscopic incisionalhernia repair in general surgery practice: early outcomes in an unselected series of patients. [SurgLaparoscEndoscPercutan Tech](http://www.ncbi.nlm.nih.gov/pubmed?term=bamehriz%20incisional%20hernia) 14: 207-9. **(4)**
165. [Motson RW](http://www.ncbi.nlm.nih.gov/pubmed?term=Motson%20RW%5BAuthor%5D&cauthor=true&cauthor_uid=17048281), [Engledow AH](http://www.ncbi.nlm.nih.gov/pubmed?term=Engledow%20AH%5BAuthor%5D&cauthor=true&cauthor_uid=17048281), [Medhurst C](http://www.ncbi.nlm.nih.gov/pubmed?term=Medhurst%20C%5BAuthor%5D&cauthor=true&cauthor_uid=17048281), [Adib R](http://www.ncbi.nlm.nih.gov/pubmed?term=Adib%20R%5BAuthor%5D&cauthor=true&cauthor_uid=17048281), [Warren SJ](http://www.ncbi.nlm.nih.gov/pubmed?term=Warren%20SJ%5BAuthor%5D&cauthor=true&cauthor_uid=17048281) (2006)Laparoscopic incisionalhernia repair with a self-centring suture. [Br J Surg](http://www.ncbi.nlm.nih.gov/pubmed/17048281) 93: 1549-53. **(4)**
166. [Raftopoulos I](http://www.ncbi.nlm.nih.gov/pubmed?term=Raftopoulos%20I%5BAuthor%5D&cauthor=true&cauthor_uid=17522922), [Courcoulas AP](http://www.ncbi.nlm.nih.gov/pubmed?term=Courcoulas%20AP%5BAuthor%5D&cauthor=true&cauthor_uid=17522922) (2007)Outcome of laparoscopic ventral hernia repair in morbidly obese patients with a body mass index exceeding 35 kg/m2. [SurgEndosc](http://www.ncbi.nlm.nih.gov/pubmed/17522922) 21: 2293-7. **(4)**
167. Wassenaar EB, Raymakers JTFJ, Rakic S (2008) Impact of the mesh fixation technique on operative time in laparoscopic repair of ventral hernias. Hernia 12: 23-25. **(4)**
168. [Berrevoet F](http://www.ncbi.nlm.nih.gov/pubmed?term=%22Berrevoet%20F%22%5BAuthor%5D), [Fierens K](http://www.ncbi.nlm.nih.gov/pubmed?term=%22Fierens%20K%22%5BAuthor%5D), [De Gols J](http://www.ncbi.nlm.nih.gov/pubmed?term=%22De%20Gols%20J%22%5BAuthor%5D), [Navez B](http://www.ncbi.nlm.nih.gov/pubmed?term=%22Navez%20B%22%5BAuthor%5D), [Van Bastelaere W](http://www.ncbi.nlm.nih.gov/pubmed?term=%22Van%20Bastelaere%20W%22%5BAuthor%5D), [Meir E](http://www.ncbi.nlm.nih.gov/pubmed?term=%22Meir%20E%22%5BAuthor%5D), [Ceulemans R](http://www.ncbi.nlm.nih.gov/pubmed?term=%22Ceulemans%20R%22%5BAuthor%5D) (2009)Multicentric observational cohort study evaluating a composite mesh with incorporated oxidized regenerated cellulose in laparoscopic ventral hernia repair. [Hernia](http://www.ncbi.nlm.nih.gov/pubmed/18682886) 13:23-7.**(4)**
169. [Edwards C](http://www.ncbi.nlm.nih.gov/pubmed?term=Edwards%20C%5BAuthor%5D&cauthor=true&cauthor_uid=19462203), [Geiger T](http://www.ncbi.nlm.nih.gov/pubmed?term=Geiger%20T%5BAuthor%5D&cauthor=true&cauthor_uid=19462203), [Bartow K](http://www.ncbi.nlm.nih.gov/pubmed?term=Bartow%20K%5BAuthor%5D&cauthor=true&cauthor_uid=19462203), [Ramaswamy A](http://www.ncbi.nlm.nih.gov/pubmed?term=Ramaswamy%20A%5BAuthor%5D&cauthor=true&cauthor_uid=19462203), [Fearing N](http://www.ncbi.nlm.nih.gov/pubmed?term=Fearing%20N%5BAuthor%5D&cauthor=true&cauthor_uid=19462203), [Thaler K](http://www.ncbi.nlm.nih.gov/pubmed?term=Thaler%20K%5BAuthor%5D&cauthor=true&cauthor_uid=19462203), [Ramshaw B](http://www.ncbi.nlm.nih.gov/pubmed?term=Ramshaw%20B%5BAuthor%5D&cauthor=true&cauthor_uid=19462203) (2009) Laparoscopic transperitoneal repair of flank hernias: a retrospective review of 27 patients.[SurgEndosc](http://www.ncbi.nlm.nih.gov/pubmed?term=Laparoscopic%20transperitoneal%20repair%20of%20flank%20hernias%3A%20a%20retrospective%20review%20of%2027%20patients) 23: 2692-6. **(4)**
170. [Moreno-Egea A](http://www.ncbi.nlm.nih.gov/pubmed?term=Moreno-Egea%20A%5BAuthor%5D&cauthor=true&cauthor_uid=18574413), [Cartagena J](http://www.ncbi.nlm.nih.gov/pubmed?term=Cartagena%20J%5BAuthor%5D&cauthor=true&cauthor_uid=18574413), [Vicente JP](http://www.ncbi.nlm.nih.gov/pubmed?term=Vicente%20JP%5BAuthor%5D&cauthor=true&cauthor_uid=18574413), [Carrillo A](http://www.ncbi.nlm.nih.gov/pubmed?term=Carrillo%20A%5BAuthor%5D&cauthor=true&cauthor_uid=18574413), [Aguayo JL](http://www.ncbi.nlm.nih.gov/pubmed?term=Aguayo%20JL%5BAuthor%5D&cauthor=true&cauthor_uid=18574413) (2008)Laparoscopic incisionalhernia repair as a day surgery procedure: audit of 127 consecutive cases in a university hospital. [SurgLaparoscEndoscPercutan Tech](http://www.ncbi.nlm.nih.gov/pubmed/18574413) 18: 267-71. **(4)**
171. [Olmi S](http://www.ncbi.nlm.nih.gov/pubmed?term=Olmi%20S%5BAuthor%5D&cauthor=true&cauthor_uid=20932376), [Cesana G](http://www.ncbi.nlm.nih.gov/pubmed?term=Cesana%20G%5BAuthor%5D&cauthor=true&cauthor_uid=20932376), [Sagutti L](http://www.ncbi.nlm.nih.gov/pubmed?term=Sagutti%20L%5BAuthor%5D&cauthor=true&cauthor_uid=20932376), [Pagano C](http://www.ncbi.nlm.nih.gov/pubmed?term=Pagano%20C%5BAuthor%5D&cauthor=true&cauthor_uid=20932376), [Vittoria G](http://www.ncbi.nlm.nih.gov/pubmed?term=Vittoria%20G%5BAuthor%5D&cauthor=true&cauthor_uid=20932376), [Croce E](http://www.ncbi.nlm.nih.gov/pubmed?term=Croce%20E%5BAuthor%5D&cauthor=true&cauthor_uid=20932376) (2010)Laparoscopic incisionalhernia repair with fibrin glue in select patients. [JSLS](http://www.ncbi.nlm.nih.gov/pubmed/20932376) 14: 240-5. **(4)**
172. SturtNJH,Christopher CL, Engledow AH, Menzies D, Motson R (2011) Results of Laparoscopic Repair of Primary and Recurrent Incisional Hernias at a Single UK Institution. SurgLaparoscEndoscPercutan Tech 21:86–89. **(4)**
173. [Engledow AH](http://www.ncbi.nlm.nih.gov/pubmed?term=Engledow%20AH%5BAuthor%5D&cauthor=true&cauthor_uid=17111283), [Sengupta N](http://www.ncbi.nlm.nih.gov/pubmed?term=Sengupta%20N%5BAuthor%5D&cauthor=true&cauthor_uid=17111283), [Akhras F](http://www.ncbi.nlm.nih.gov/pubmed?term=Akhras%20F%5BAuthor%5D&cauthor=true&cauthor_uid=17111283), [Tutton M](http://www.ncbi.nlm.nih.gov/pubmed?term=Tutton%20M%5BAuthor%5D&cauthor=true&cauthor_uid=17111283), [Warren SJ](http://www.ncbi.nlm.nih.gov/pubmed?term=Warren%20SJ%5BAuthor%5D&cauthor=true&cauthor_uid=17111283) (2007) Day case laparoscopic incisional hernia repair is feasible, acceptable, and cost effective.[SurgEndosc](http://www.ncbi.nlm.nih.gov/pubmed/17111283) 21:84-6. **(4)**
174. [Wright BE](http://www.ncbi.nlm.nih.gov/pubmed?term=Wright%20BE%5BAuthor%5D&cauthor=true&cauthor_uid=11893110), [Niskanen BD](http://www.ncbi.nlm.nih.gov/pubmed?term=Niskanen%20BD%5BAuthor%5D&cauthor=true&cauthor_uid=11893110), [Peterson DJ](http://www.ncbi.nlm.nih.gov/pubmed?term=Peterson%20DJ%5BAuthor%5D&cauthor=true&cauthor_uid=11893110), [Ney AL](http://www.ncbi.nlm.nih.gov/pubmed?term=Ney%20AL%5BAuthor%5D&cauthor=true&cauthor_uid=11893110), [Odland MD](http://www.ncbi.nlm.nih.gov/pubmed?term=Odland%20MD%5BAuthor%5D&cauthor=true&cauthor_uid=11893110), [VanCamp J](http://www.ncbi.nlm.nih.gov/pubmed?term=VanCamp%20J%5BAuthor%5D&cauthor=true&cauthor_uid=11893110), [Zera RT](http://www.ncbi.nlm.nih.gov/pubmed?term=Zera%20RT%5BAuthor%5D&cauthor=true&cauthor_uid=11893110), [Rodriguez JL](http://www.ncbi.nlm.nih.gov/pubmed?term=Rodriguez%20JL%5BAuthor%5D&cauthor=true&cauthor_uid=11893110) (2002) Laparoscopic ventral hernia repair: are there comparative advantages over traditional methods of repair?[Am Surg](http://www.ncbi.nlm.nih.gov/pubmed/11893110) 68:291-5. **(3)**
175. [Alkhoury F](http://www.ncbi.nlm.nih.gov/pubmed?term=Alkhoury%20F%5BAuthor%5D&cauthor=true&cauthor_uid=21471797), [Helton S](http://www.ncbi.nlm.nih.gov/pubmed?term=Helton%20S%5BAuthor%5D&cauthor=true&cauthor_uid=21471797), [Ippolito RJ](http://www.ncbi.nlm.nih.gov/pubmed?term=Ippolito%20RJ%5BAuthor%5D&cauthor=true&cauthor_uid=21471797) (2011) Cost and clinical outcomes of laparoscopic ventral hernia repair using intraperitoneal non heavyweight polypropylene mesh.[Surg Laparosc Endosc Percutan Tech](http://www.ncbi.nlm.nih.gov/pubmed/21471797) 21: 82-5. **(3)**
176. [Mussack T](http://www.ncbi.nlm.nih.gov/pubmed?term=Mussack%20T%5BAuthor%5D&cauthor=true&cauthor_uid=16424985), [Ladurner R](http://www.ncbi.nlm.nih.gov/pubmed?term=Ladurner%20R%5BAuthor%5D&cauthor=true&cauthor_uid=16424985), [Vogel T](http://www.ncbi.nlm.nih.gov/pubmed?term=Vogel%20T%5BAuthor%5D&cauthor=true&cauthor_uid=16424985), [Lienemann A](http://www.ncbi.nlm.nih.gov/pubmed?term=Lienemann%20A%5BAuthor%5D&cauthor=true&cauthor_uid=16424985), [Eder-Willwohl A](http://www.ncbi.nlm.nih.gov/pubmed?term=Eder-Willwohl%20A%5BAuthor%5D&cauthor=true&cauthor_uid=16424985), [Hallfeldt KK](http://www.ncbi.nlm.nih.gov/pubmed?term=Hallfeldt%20KK%5BAuthor%5D&cauthor=true&cauthor_uid=16424985) (2006) Health-related quality-of-life changes after laparoscopic and open incisional hernia repair: a matched pair analysis. [Surg Endosc](http://www.ncbi.nlm.nih.gov/pubmed?term=Surg%20Endosc%20%282006%29%2020%3A%20410%E2%80%93413) 20: 410-3. **(3)**
177. Wassenaar E, Schoenmaeckers E, Raymakers J, van der Palen J (2010) Mesh-fixation method and pain and quality of life after laparoscopic ventral or incisional hernia repair: a randomized trial of three fixation techniques. Surg Endosc 24:1296-302. **(1b)**
178. [Cobb WS](http://www.ncbi.nlm.nih.gov/pubmed?term=%22Cobb%20WS%22%5BAuthor%5D), [Kercher KW](http://www.ncbi.nlm.nih.gov/pubmed?term=%22Kercher%20KW%22%5BAuthor%5D), [Heniford BT](http://www.ncbi.nlm.nih.gov/pubmed?term=%22Heniford%20BT%22%5BAuthor%5D) (2005) Laparoscopic repair of incisional hernias. [Surg Clin North Am](http://www.ncbi.nlm.nih.gov/pubmed/15619531) 85:91-103. **(5)**
179. [Cassar K](http://www.ncbi.nlm.nih.gov/pubmed?term=Cassar%20K%5BAuthor%5D&cauthor=true&cauthor_uid=11972542), [Munro A](http://www.ncbi.nlm.nih.gov/pubmed?term=Munro%20A%5BAuthor%5D&cauthor=true&cauthor_uid=11972542) (2002) Surgical treatment of incisional hernia.[Br J Surg](http://www.ncbi.nlm.nih.gov/pubmed/11972542)89: 534-45**. (2a)**
180. Bower CE, Reade CC, Kirby LW, Roth JS (2004) Complications of laparoscopic incisional-ventral hernia repair: the experience of a single institution. Surg Endosc 18: 672–675. **(4)**
181. Bedi AP, Bhatti T, Amin A, Zuberi J (2007) Laparoscopic incisional and ventral hernia repair. J Minim Access Surg 3:83-90. **(2a)**
182. [McKinlay RD](http://www.ncbi.nlm.nih.gov/pubmed?term=%22McKinlay%20RD%22%5BAuthor%5D), [Park A](http://www.ncbi.nlm.nih.gov/pubmed?term=%22Park%20A%22%5BAuthor%5D) (2004)Laparoscopic ventral incisional hernia repair: a more effective alternative to conventional repair of recurrent incisional hernia.[J Gastrointest Surg](http://www.ncbi.nlm.nih.gov/pubmed/15358326) 8:670-4. **(2b)**
183. [LeBlanc KA](http://www.ncbi.nlm.nih.gov/pubmed?term=LeBlanc%20KA%5BAuthor%5D&cauthor=true&cauthor_uid=17287923) (2007) Laparoscopic incisional hernia repair: are transfascial sutures necessary? A review of the literature.[Surg Endosc](http://www.ncbi.nlm.nih.gov/pubmed/17287923) 21: 508-13. **(2a)**
184. [Rudmik LR](http://www.ncbi.nlm.nih.gov/pubmed?term=Rudmik%20LR%5BAuthor%5D&cauthor=true&cauthor_uid=16453075), [Schieman C](http://www.ncbi.nlm.nih.gov/pubmed?term=Schieman%20C%5BAuthor%5D&cauthor=true&cauthor_uid=16453075), [Dixon E](http://www.ncbi.nlm.nih.gov/pubmed?term=Dixon%20E%5BAuthor%5D&cauthor=true&cauthor_uid=16453075), [Debru E](http://www.ncbi.nlm.nih.gov/pubmed?term=Debru%20E%5BAuthor%5D&cauthor=true&cauthor_uid=16453075) (2006) Laparoscopic incisional hernia repair: a review of the literature.[Hernia](http://www.ncbi.nlm.nih.gov/pubmed?term=Rudmik%20LR%20ventral%20hernia) 10: 110-9. **(2a)**
185. [Ching SS](http://www.ncbi.nlm.nih.gov/pubmed?term=Ching%20SS%5BAuthor%5D&cauthor=true&cauthor_uid=18622552), [Sarela AI](http://www.ncbi.nlm.nih.gov/pubmed?term=Sarela%20AI%5BAuthor%5D&cauthor=true&cauthor_uid=18622552), [Dexter SP](http://www.ncbi.nlm.nih.gov/pubmed?term=Dexter%20SP%5BAuthor%5D&cauthor=true&cauthor_uid=18622552), [Hayden JD](http://www.ncbi.nlm.nih.gov/pubmed?term=Hayden%20JD%5BAuthor%5D&cauthor=true&cauthor_uid=18622552), [McMahon MJ](http://www.ncbi.nlm.nih.gov/pubmed?term=McMahon%20MJ%5BAuthor%5D&cauthor=true&cauthor_uid=18622552) (2008) Comparison of early outcomes for laparoscopic ventral hernia repair between nonobese and morbidly obese patient populations.[Surg Endosc](http://www.ncbi.nlm.nih.gov/pubmed/18622552) 22: 2244-50. **(2b)**
186. [Ceccarelli G](http://www.ncbi.nlm.nih.gov/pubmed?term=Ceccarelli%20G%5BAuthor%5D&cauthor=true&cauthor_uid=17623245), [Casciola L](http://www.ncbi.nlm.nih.gov/pubmed?term=Casciola%20L%5BAuthor%5D&cauthor=true&cauthor_uid=17623245), [Pisanelli MC](http://www.ncbi.nlm.nih.gov/pubmed?term=Pisanelli%20MC%5BAuthor%5D&cauthor=true&cauthor_uid=17623245), [Bartoli A](http://www.ncbi.nlm.nih.gov/pubmed?term=Bartoli%20A%5BAuthor%5D&cauthor=true&cauthor_uid=17623245), [Di Zitti L](http://www.ncbi.nlm.nih.gov/pubmed?term=Di%20Zitti%20L%5BAuthor%5D&cauthor=true&cauthor_uid=17623245), [Spaziani A](http://www.ncbi.nlm.nih.gov/pubmed?term=Spaziani%20A%5BAuthor%5D&cauthor=true&cauthor_uid=17623245), [Biancafarina A](http://www.ncbi.nlm.nih.gov/pubmed?term=Biancafarina%20A%5BAuthor%5D&cauthor=true&cauthor_uid=17623245), [Stefanoni M](http://www.ncbi.nlm.nih.gov/pubmed?term=Stefanoni%20M%5BAuthor%5D&cauthor=true&cauthor_uid=17623245), [Patriti A](http://www.ncbi.nlm.nih.gov/pubmed?term=Patriti%20A%5BAuthor%5D&cauthor=true&cauthor_uid=17623245)(2008) Comparing fibrin sealant with staples for mesh fixation in laparoscopic transabdominal hernia repair: a case control-study. [Surg Endosc](http://www.ncbi.nlm.nih.gov/pubmed?term=Surg%20Endosc%20%282008%29%2022%3A668%E2%80%93673)22: 668-73. **(2b)**
187. Chowbey PK, Sharma A, Khullar R Mann V, Baijal M, Vashistha A (2000) Laparoscopic ventral hernia repair. J Laparoendosc Adv Surg.Tech A 10: 79–84. **(4)**
188. Binnebosel M, Rosch R, Junge K, Flanagan TC, Schwab R, Schumpelick V, Klinge U (2007) Biochemical analysis of overlap and mesh dislocation in an incisional hernia model in vitro. Surgery 142: 365-71. **(4)**
189. [Verbo A](http://www.ncbi.nlm.nih.gov/pubmed?term=Verbo%20A%5BAuthor%5D&cauthor=true&cauthor_uid=17907969), [Petito L](http://www.ncbi.nlm.nih.gov/pubmed?term=Petito%20L%5BAuthor%5D&cauthor=true&cauthor_uid=17907969), [Manno A](http://www.ncbi.nlm.nih.gov/pubmed?term=Manno%20A%5BAuthor%5D&cauthor=true&cauthor_uid=17907969), [Coco C](http://www.ncbi.nlm.nih.gov/pubmed?term=Coco%20C%5BAuthor%5D&cauthor=true&cauthor_uid=17907969), [Mattana C](http://www.ncbi.nlm.nih.gov/pubmed?term=Mattana%20C%5BAuthor%5D&cauthor=true&cauthor_uid=17907969), [Lurati M](http://www.ncbi.nlm.nih.gov/pubmed?term=Lurati%20M%5BAuthor%5D&cauthor=true&cauthor_uid=17907969), [Pedretti G](http://www.ncbi.nlm.nih.gov/pubmed?term=Pedretti%20G%5BAuthor%5D&cauthor=true&cauthor_uid=17907969), [Rizzo G](http://www.ncbi.nlm.nih.gov/pubmed?term=Rizzo%20G%5BAuthor%5D&cauthor=true&cauthor_uid=17907969), [Sermoneta D](http://www.ncbi.nlm.nih.gov/pubmed?term=Sermoneta%20D%5BAuthor%5D&cauthor=true&cauthor_uid=17907969), [Lodoli C](http://www.ncbi.nlm.nih.gov/pubmed?term=Lodoli%20C%5BAuthor%5D&cauthor=true&cauthor_uid=17907969), [Nunziata J](http://www.ncbi.nlm.nih.gov/pubmed?term=Nunziata%20J%5BAuthor%5D&cauthor=true&cauthor_uid=17907969), [D'Ugo D](http://www.ncbi.nlm.nih.gov/pubmed?term=D%27Ugo%20D%5BAuthor%5D&cauthor=true&cauthor_uid=17907969) (2007) Laparoscopic approach to recurrent incisional hernia repair: a 3-year experience.[J Laparoendosc Adv Surg Tech A](http://www.ncbi.nlm.nih.gov/pubmed/17907969) 17:591-5. **(4)**
190. [Theodoropoulou K](http://www.ncbi.nlm.nih.gov/pubmed?term=Theodoropoulou%20K%5BAuthor%5D&cauthor=true&cauthor_uid=20529535), [Lethaby D](http://www.ncbi.nlm.nih.gov/pubmed?term=Lethaby%20D%5BAuthor%5D&cauthor=true&cauthor_uid=20529535), [Hill J](http://www.ncbi.nlm.nih.gov/pubmed?term=Hill%20J%5BAuthor%5D&cauthor=true&cauthor_uid=20529535), [Gupta S](http://www.ncbi.nlm.nih.gov/pubmed?term=Gupta%20S%5BAuthor%5D&cauthor=true&cauthor_uid=20529535), [Bradpiece H](http://www.ncbi.nlm.nih.gov/pubmed?term=Bradpiece%20H%5BAuthor%5D&cauthor=true&cauthor_uid=20529535) (2010) Laparoscopic hernia repair: a two-port technique.[JSLS](http://www.ncbi.nlm.nih.gov/pubmed?term=Theodoropoulou%202010%20incisional%20hernia) 14: 103-5. **(4)**
191. [Costanza MJ](http://www.ncbi.nlm.nih.gov/pubmed?term=Costanza%20MJ%5BAuthor%5D&cauthor=true&cauthor_uid=9843329), [Heniford BT](http://www.ncbi.nlm.nih.gov/pubmed?term=Heniford%20BT%5BAuthor%5D&cauthor=true&cauthor_uid=9843329), [Arca MJ](http://www.ncbi.nlm.nih.gov/pubmed?term=Arca%20MJ%5BAuthor%5D&cauthor=true&cauthor_uid=9843329), [Mayes JT](http://www.ncbi.nlm.nih.gov/pubmed?term=Mayes%20JT%5BAuthor%5D&cauthor=true&cauthor_uid=9843329), [Gagner M](http://www.ncbi.nlm.nih.gov/pubmed?term=Gagner%20M%5BAuthor%5D&cauthor=true&cauthor_uid=9843329) (1998) Laparoscopic repair of recurrent ventral hernias.[Am Surg](http://www.ncbi.nlm.nih.gov/pubmed/9843329)64: 1121-5. **(4)**
192. [Szymanski J](http://www.ncbi.nlm.nih.gov/pubmed?term=Szymanski%20J%5BAuthor%5D&cauthor=true&cauthor_uid=10890970), [Voitk A](http://www.ncbi.nlm.nih.gov/pubmed?term=Voitk%20A%5BAuthor%5D&cauthor=true&cauthor_uid=10890970), [Joffe J](http://www.ncbi.nlm.nih.gov/pubmed?term=Joffe%20J%5BAuthor%5D&cauthor=true&cauthor_uid=10890970), [Alvarez C](http://www.ncbi.nlm.nih.gov/pubmed?term=Alvarez%20C%5BAuthor%5D&cauthor=true&cauthor_uid=10890970), [Rosenthal G](http://www.ncbi.nlm.nih.gov/pubmed?term=Rosenthal%20G%5BAuthor%5D&cauthor=true&cauthor_uid=10890970) (2000)Technique and early results of outpatient laparoscopic mesh onlay repair of ventral hernias. [Surg Endosc](http://www.ncbi.nlm.nih.gov/pubmed/10890970) 14:582-4. **(4)**
193. [Gillian GK](http://www.ncbi.nlm.nih.gov/pubmed?term=Gillian%20GK%5BAuthor%5D&cauthor=true&cauthor_uid=12500829), [Geis WP](http://www.ncbi.nlm.nih.gov/pubmed?term=Geis%20WP%5BAuthor%5D&cauthor=true&cauthor_uid=12500829), [Grover G](http://www.ncbi.nlm.nih.gov/pubmed?term=Grover%20G%5BAuthor%5D&cauthor=true&cauthor_uid=12500829) (2002) Laparoscopic incisional and ventral hernia repair (LIVH): an evolving outpatient technique. [JSLS](http://www.ncbi.nlm.nih.gov/pubmed?term=gillian%20ventral%20hernia) 6:315-22. **(4)**
194. [Eitan A](http://www.ncbi.nlm.nih.gov/pubmed?term=Eitan%20A%5BAuthor%5D&cauthor=true&cauthor_uid=12470403), [Bickel A](http://www.ncbi.nlm.nih.gov/pubmed?term=Bickel%20A%5BAuthor%5D&cauthor=true&cauthor_uid=12470403) (2002)Laparoscopically assisted approach for postoperative ventral hernia repair. [J Laparoendosc Adv Surg Tech A](http://www.ncbi.nlm.nih.gov/pubmed/12470403) 12:309-11. **(4)**
195. [Moreno-Egea A](http://www.ncbi.nlm.nih.gov/pubmed?term=Moreno-Egea%20A%5BAuthor%5D&cauthor=true&cauthor_uid=19533233), [Bustos JA](http://www.ncbi.nlm.nih.gov/pubmed?term=Bustos%20JA%5BAuthor%5D&cauthor=true&cauthor_uid=19533233), [Girela E](http://www.ncbi.nlm.nih.gov/pubmed?term=Girela%20E%5BAuthor%5D&cauthor=true&cauthor_uid=19533233), [Aguayo-Albasini JL](http://www.ncbi.nlm.nih.gov/pubmed?term=Aguayo-Albasini%20JL%5BAuthor%5D&cauthor=true&cauthor_uid=19533233) (2010)Long-term results of laparoscopic repair of incisional hernias using an intraperitoneal composite mesh. [SurgEndosc](http://www.ncbi.nlm.nih.gov/pubmed/19533233) 24: 359-65. **(4)**
196. Frantzides CT, Carlson MA, Zografakis JG, Madan AK, Moore RE (2004) Minimally invasive incisional herniorrhaphy: a review of 208 cases. SurgEndosc 18: 1488–1491.**(4)**
